# Scalable Multiparametric Characterization of Aptamer–Target Interactions

**DOI:** 10.1021/acsnano.5c19596

**Published:** 2026-01-08

**Authors:** Marc Sulliger, Matthew Peters, Andrea Sottini, Annina Stuber, Kyungae Yang, Nako Nakatsuka, Jaime Ortega Arroyo, Romain Quidant

**Affiliations:** † Nanophotonic Systems Laboratory, Department of Mechanical and Process Engineering, 111842 ETH Zurich, 8092 Zurich, Switzerland; ‡ Laboratory of Biosensors and Bioelectronics, Department of Information Technology and Electrical Engineering, 111842 ETH Zurich, 8092 Zurich, Switzerland; § Laboratory of Chemical Nanotechnology, EPFL, 1202 Genève, Switzerland; ∥ Department of Medicine, Columbia University Irving Medical Center, New York, New York 10032, United States

**Keywords:** structure-switching aptamers, droplet microfluidics, biosensing, Förster resonance energy transfer, hyperspectral imaging, serotonin, reaction kinetics

## Abstract

Structure-switching aptamers transduce target-induced conformational changes into detectable signals, enabling the specific detection of small molecules with limited surface area and charge. Understanding these structural transitions is critical for the rational design of aptamers in downstream biosensing. However, current methods lack the scalability and high spatiotemporal resolution to characterize and resolve these structural dynamics within a single unified platform. Here, we report a scalable droplet microfluidic platform that fills this technological gap by integrating Förster resonance energy transfer with automated imaging for the multiparametric profiling of aptamer–target interactions. This integrated system enables the detailed analysis of aptamer–target interactions in picoliter volumes under physiologically relevant conditions across the millisecond-to-hour time scales. Investigating serotonin aptamers with varying stem lengths, we systematically explore structure–function relationships and translate molecular-level insights into the application-driven selection of optimal candidates. By bridging low-throughput structural characterization with a rapid, low-volume, and multiparametric readout, our platform overcomes a key barrier in translational biosensor development and lays the foundation for data-driven engineering of structure-switching aptamers tailored for diagnostics and beyond.

## Graphical abstract

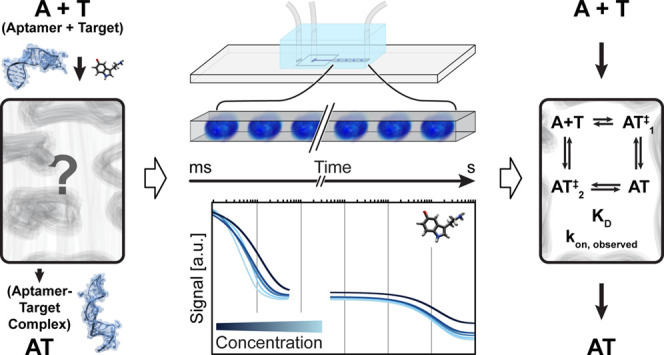

Aptamers, single-stranded oligonucleotides engineered for molecular recognition,^1,2^ are broadly used in both molecular detection and therapeutics.^3^ Generated de novo through an in vitro selection method termed systematic evolution of ligands by exponential enrichment (SELEX), aptamers provide distinct advantages as biorecognition elements in diagnostics.^4^ The SELEX process can simulate environments that closely resemble the final application, while counter-selection against nontargets with structural similarities to intended targets can improve the selectivity of candidate sequences.^5^ Aptamers are strategic for detecting small-molecule targets, where traditional protein-based bioreceptors, such as antibodies, face challenges due to the limited functional groups on small molecules, which can lead to cross-reactivity.^6^ Moreover, small molecules typically have low mass and charge, making it difficult for conventional biosensing techniques to transduce the binding event. This challenge is amplified in complex biological systems, where high concentrations of nonspecific interferents—often significantly larger in size, charge, and concentration—obscure the detection of specific analyte binding.^7^

To overcome this challenge, aptamers that undergo conformational rearrangements upon target recognition have been developed.^2,8–11^ These structural shifts amplify signal transduction by repositioning the negatively charged oligonucleotide backbone while adding an extra layer of selectivity by occurring only in the presence of the specific analyte. However, aptamers alone are prone to nonspecific interactions due to their propensity for hydrophobic interactions^12^ combined with their highly negative charge.^7^ Integrating aptamers into sensor configurations designed to mitigate interferents has enabled biosensing in complex biological systems.^13–19^ Nevertheless, developing a functional aptamer-based biosensor is inherently complex and requires careful tuning to meet specific performance criteria, such as temporal dynamics, sensitivity, and detection limits. Optimizing parameters requires comprehensive characterization of key biophysical properties, including the dissociation constant (*K*_D_) and binding kinetics of aptamer–target interactions.^2,20^

While structure–function relationships are pivotal in aptamer engineering, their characterization remains challenging. Traditional methods, such as electrophoretic mobility shift assays, surface plasmon resonance, and isothermal titration calorimetry, provide valuable thermodynamic and kinetic insights but often lack the resolution needed to fully capture the complex structural dynamics of aptamers. Solution-based spectroscopic techniques, including circular dichroism (CD)^21^ and nuclear magnetic resonance (NMR),^22^ as well as surface-based techniques, such as quartz crystal microbalance with dissipation monitoring (QCM-D),^23^ can monitor these conformational changes upon target binding. However, these approaches typically require substantial volumes and high concentrations of aptamers, which can be costly for fundamental studies involving extensive condition screening.

Flow-induced dispersion analysis (FIDA) is an alternative low-volume (nanoliter), high-throughput technique used to observe the hydrodynamic radius change upon target binding to a fluorescently labeled aptamer.^24^ However, FIDA provides only an indirect measure of conformational changes and lacks the structural resolution needed to track aptamer backbone rearrangements or reveal detailed binding modes and secondary structure transitions.^25^ In contrast, Förster resonance energy transfer (FRET) is an approach that enables direct observation of structural transitions, providing molecular-level insights into aptamer binding dynamics.^26,27^ As a nanoscale distance-dependent phenomenon, FRET relies on energy transfer between a donor and acceptor fluorophore positioned within nanometer proximity on the aptamer backbone. Aptamer conformational changes modulate the distance between the fluorophores, altering energy transfer efficiency and resulting in fluorescence signals that visually transduce the transition between target-bound and unbound states.^28,29^ Surface-based FRET approaches integrated with sequence library generation enable high-throughput abilities. However, these approaches rely on immobilization, which can perturb aptamer conformations and lack the temporal resolution needed to capture rapid aptamer-target dynamics in the ms regime.^29,30^

Further, leveraging FRET to characterize aptamer conformational dynamics demands careful design. Efficient energy transfer requires donor and acceptor fluorophores to be positioned within a few nanometers of each other^31^ in one conformational state while ensuring that their presence does not interfere with target binding. Designing such FRET-based aptamer sensors is challenging, often relying on trial-and-error due to the lack of systematic multiparametric characterization methods. Moreover, the low-throughput and labor-intensive nature of single-molecule FRET, combined with the high cost of double-fluorophore-labeled sequences, further complicates the iterative optimization process.^32^ Further, aptamer binding events do not always follow simple single-state models, as shown by recent studies using single-molecule field-effect transistor platforms.^33^ While these measurements offer valuable insights into individual binding events, their diffusion-limited nature often necessitates long observation times to detect single-molecule binding events, making them less practical for high-throughput applications.^34,35^ These limitations underscore the need for scalable, multiparametric approaches capable of capturing heterogeneous kinetic behavior.

To bridge this gap, we introduce a droplet-based read-out platform capable of tracking aptamer–target interactions using FRET-based detection in picoliter volumes with automated imaging that enables the assessment of hundreds of readouts per minute. This approach allows for real-time monitoring of aptamer binding and structural switching with high spatiotemporal resolution (∼1.49 μm, 750 μs). The ability to analyze binding kinetics with minimal reagent consumption (∼*N*_Droplets_ × 60 pL) is particularly advantageous given the high cost of fluorescently labeled aptamers. Moreover, the platform allows for direct screening of fluorescent aptamer sensors under physiologically relevant conditions (e.g., complex biological media such as serum), where competing interactions and nonspecific binding can significantly alter aptamer behavior, overcoming a critical limitation in current aptamer characterization workflows. By demonstrating the potential to characterize aptamer sensors directly in environments that reflect their intended deployment conditions, we strengthened the translational relevance of the screening results.

In this work, we demonstrate a scalable solution for multiparametric aptamer characterization. Beyond applications in FRET sensor development and characterization, the ability to systematically investigate aptamer conformational dynamics offers new opportunities for tailoring molecular recognition elements across a wide range of sensing technologies. This work establishes a foundation for more predictive, data-driven approaches in aptamer engineering, bridging the gap between fundamental biophysical studies and practical biosensing applications.

## Results and Discussion

To validate the droplet-based platform for fast and low-volume multiparametric characterization of aptamer–target interactions, we selected a serotonin-specific aptamer as the model system. This sequence has been extensively characterized through a range of experimental^8,19^ and computational studies,^17,36^ providing a reliable benchmark with validated binding behavior and conformational dynamics. Further, recent single-molecule transistor studies have suggested a multistep binding mechanism for this serotonin aptamer, characterized by distinct kinetic components,^33^ which we sought to investigate further using our hyperspectral imaging platform with high temporal resolution.

To modulate the binding dynamics of the serotonin aptamer, we designed variants (L3, L4, and L5) that differ only in stem length, as stem truncation can alter folding efficiency, structural rigidity, and consequently binding and unbinding kinetics.^11,37,38^ Each sequence was site-specifically labeled with donor and acceptor FRET dyes at previously validated positions that maximize the measurable FRET response^8^ while avoiding disruption of the binding interface, enabling direct comparisons across constructs with varying stem stabilities (Figure 1a). This structural feature is critical for rational aptamer design, particularly in applications that demand precise and tunable structure-switching behavior across different time scales.

**Figure 1:**
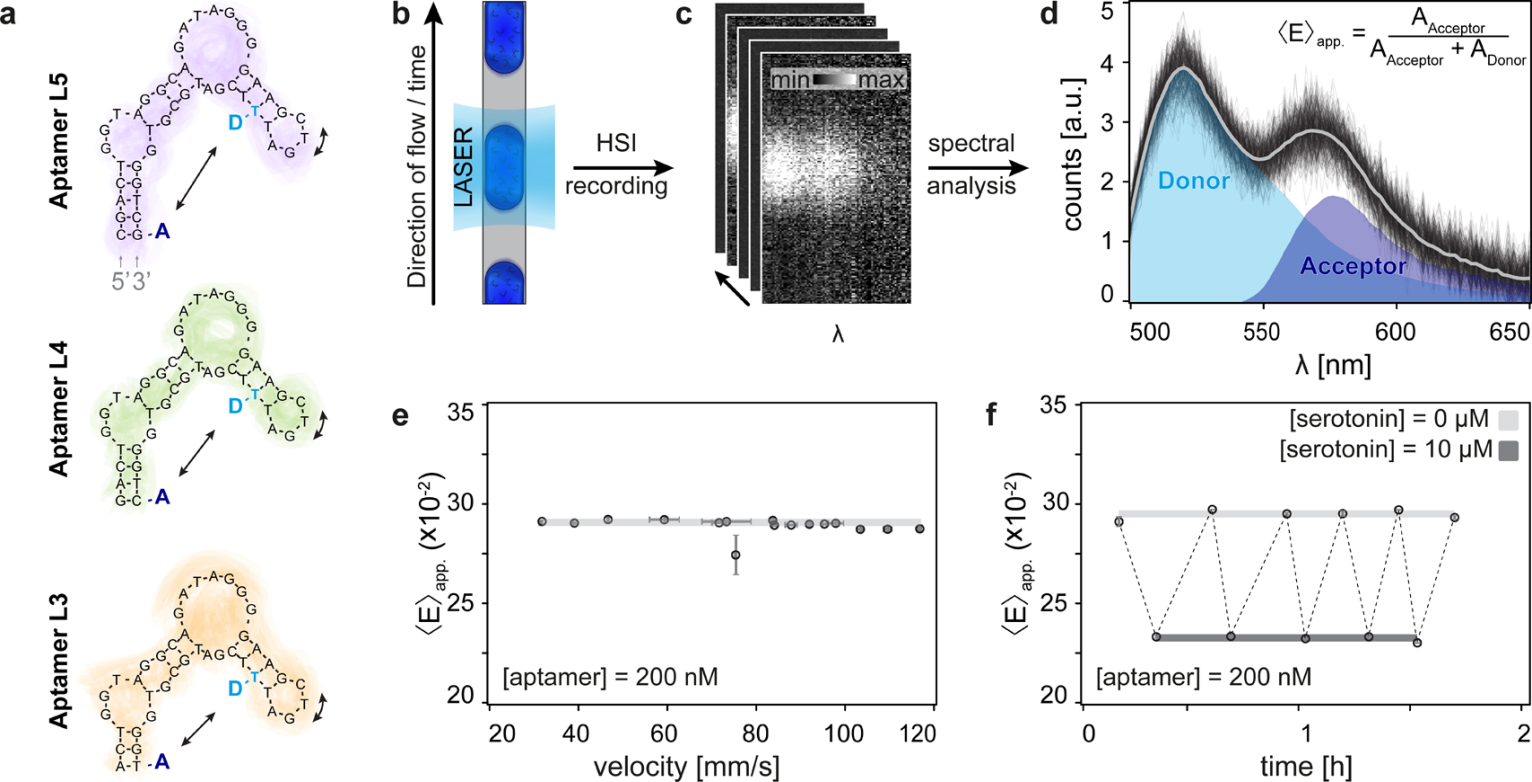
Validation of droplet-based aptamer FRET. (a) Schematic representation of the secondary structures of the investigated structure-switching serotonin aptamers with different stem lengths. From top to bottom: aptamers L5, L4, and L3. The structure of aptamer L5 was predicted using MFold, while aptamers L4 and L3 are simply shown using the same schematic notation. (b) Schematic representation of droplet-based measurement, where a narrowband source quasiconfocally illuminates a single droplet, exciting the donor fluorophores from the doubly labeled aptamer sample. (c) Representative hyperspectral images as recorded using the integrated imaging platform. (d) Background corrected emission spectra from individual droplets excited at 487 nm (black lines). Each line is an average of over 30 spectra collected in a single shot from the same droplet. The individual droplet spectra are subsequently averaged (gray line) to increase the signal-to-noise ratio (SNR). The resulting spectrum is then evaluated based on the integrated donor and acceptor intensity, which afterward allows for quantitative measurements. (e) Validation of signal stability for increasing droplet velocities. Error bars represent the STD over three measurements in the same microfluidic chip. (f) Repeated measurements for alternating conditions of no serotonin and high serotonin concentrations (10 μM). After each measurement containing serotonin, the chip was rinsed with a buffer solution. All displayed data are based on aptamer L5.

### Validation of Droplet-Based FRET Fluorescence Spectroscopy Using Hyperspectral Imaging (HSI) Platform

In this work, we used a custom-built HSI platform^39^ depicted in Figure S1 and described in detail in the Supporting Information. In brief, this platform combines a microfluidic droplet chip with a multichannel optical readout, providing simultaneous spectral and spatial information on individual droplets, alongside the measurement of droplet frequencies and spacing. Moreover, the integration of two-layer microfluidic architectures (see SI and Figure S2 for more information on the microfluidic chip and its fabrication), featuring Quake valves,^40^ provides high control over fluid manipulation even with multiple sample inputs. The choice of a droplet-based approach was driven by the need to minimize interactions with the substrate, which leads to molecular depletion and the need for passivation, while enabling rapid in-chip mixing to access fast-scale kinetics.^41^

Figure 1b–d outlines the measurement principle of our platform and its validation for FRET-based ensemble fluorescence measurements. A stabilized narrowband 487 nm LASER illuminates an area of the microfluidic chip slightly exceeding the size of a single-aptamer-containing droplet (Figure 1b), thereby exciting the donor dye on the FRET-labeled aptamers. Light emitted from each droplet is collected and imaged onto a HSI detector (Figure 1c), yielding a spectral signature (see SI and Figure S3 for more details). Fluorescence intensity spectra of individual droplets were temporally averaged to obtain a single representative spectrum prior to calculating the apparent FRET efficiency, ⟨*E*⟩_app._ (Figure 1d), defined as the ratio of acceptor emission to the total emission from FRET-labeled aptamers. The ⟨*E*⟩_app._ was calculated by fitting the spectral signature of a donor-only labeled aptamer to the averaged spectrum, allowing for the separation of individual contributions from the donor and acceptor dyes. This approach enabled monitoring of both the absolute change in donor emission intensity and the relative change in emission between the FRET pair, validating our platform for robust and quantitative data acquisition and analysis. The ⟨*E*⟩_app._ was not affected by increasing droplet velocity and hence blurring (Figure 1e).

As a subsequent validation step, the system was checked for sample crosstalk, nonspecific binding, and sample depletion that may occur between subsequent measurements of different serotonin concentrations. To this end, we measured the ⟨*E*⟩_app._ of a fixed concentration of aptamer while alternating between the absence (i.e., 0 μM) and high concentration (i.e., 10 μM) of serotonin within the same chip. This process was repeated over several cycles, each followed by a rinsing step with buffer. As shown in Figure 1f, stable and reproducible signals were observed across multiple repetitions. The absence of sample depletion or carryover confirms both the retention of performance and the repetitive-use efficiency of the droplet microfluidic platform. A series of calibration and control experiments were conducted to further validate the system, which included optimizations of aptamer concentration, exposure time, and droplet velocity, as well as evaluation of aptamer stability over time (Figure S4). Additionally, variations in focus position were shown not to affect the measured ⟨*E*⟩_app._ values (Figure S5), underscoring the reliability and reproducibility of this approach.

### Multiparametric Characterization of Aptamer Kinetics Based on Three Measurement Protocols

To obtain complementary information and access different kinetic time scales within the same droplet chip, we developed three different measurement protocols summarized in Figure 2.

**Figure 2:**
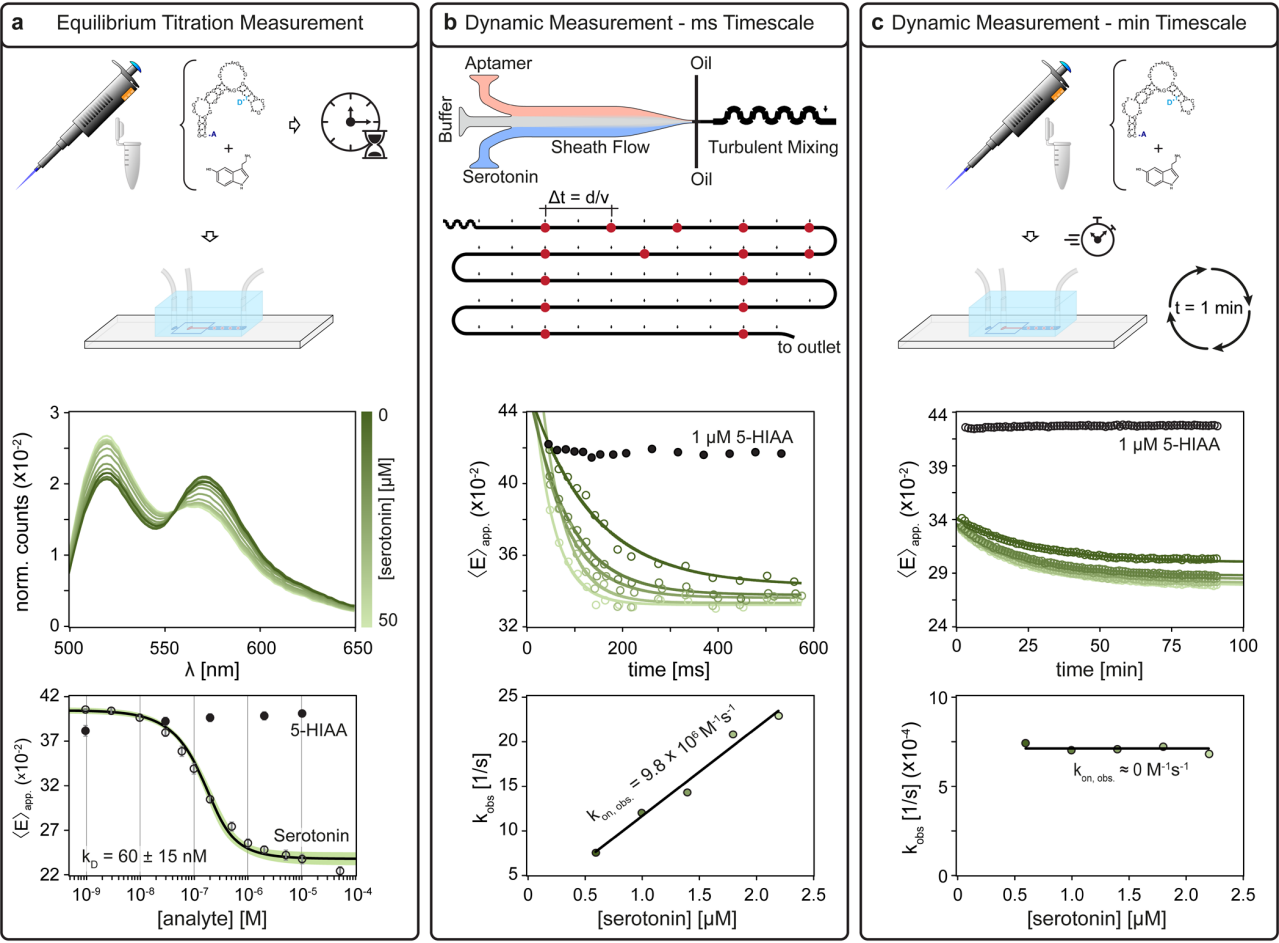
Multiparametric aptamer characterization by droplet-based FRET spectroscopy. (a) For equilibrium titration measurements, the aptamers and analytes were mixed off-chip and incubated for at least 1 h. The sample was then loaded into a droplet chip, and FRET spectra were recorded. These spectra were mapped to a sigmoidal binding model to extract the binding affinity. For dynamic measurements, two approaches were followed to probe different time scales. (b) For measurements on the millisecond time scale, the aptamer and sample were fed individually into a microfluidic chip. Inside the chip, the two phases were separated by a buffer sheath flow until the droplet was formed (i.e., *t* = 0) and chaotic mixing takes place. Afterward, a FRET measurement was performed at different positions, encoding different time points. An exponential fit describing the signal change allows for the extraction of an observed rate constant, from which the association rate (*k*_on, obs._) can be determined. (c) For time scales on the order of minutes to hours, the sample was mixed off-chip but then immediately loaded into the droplet chip. Measurements were then performed at defined intervals at a fixed position along the channel. As for the short time scale, an observed rate constant was extracted and plotted as a function of serotonin concentration to determine *k*_on, obs._.

The first protocol determines the affinity constant (*K*_D_) between the aptamer and the analyte (serotonin) at equilibrium by constructing a titration curve. Specifically, the aptamers and serotonin are mixed in a reaction tube, incubated for at least 1 h, and then loaded into droplets. The FRET spectra at each serotonin concentration are then analyzed to determine ⟨*E*⟩_app._. The resulting titration curve is fitted to a binding model (see the Materials and Methods section) to extract the *K*_D_ for the specific aptamer. To confirm binding selectivity, we tested several concentrations of a negative control 5-hydroxyindole-3-acetic acid (5-HIAA, a structurally similar serotonin metabolite), which is expected to interact negligibly with the aptamer.

The second protocol measures the fast-binding kinetics of aptamer–target interactions at time scales below 500 ms. Specifically, in this protocol, the aptamers, buffer, and serotonin (or 5-HIAA) are introduced into the droplet chip via separate inlets. Shortly before the droplet production, the three phases merge into a single channel, where they co-flow for a short time. The buffer phase, placed in between the other two, acts as a sheath flow, effectively separating aptamer and serotonin solutions until droplet formation (Figure S6). Once encapsulated into a droplet, the reagents pass through a spike-modified serpentine channel, which ensures complete sample mixing, thereby precisely controlling the start of the reaction (*t* = 0 s). This configuration enables monitoring of dynamics at the millisecond time scale with minimal dead times, overcoming a critical challenge of conventional methods.

Time evolution of the reaction is encoded spatially along the outlet channel downstream of the mixing structure, whereby time points can be precisely retrieved from the measurement location, the production frequency, and the spacing between droplets (see Supporting Information and Figure S7). The change in ⟨*E*⟩_app._ caused by aptamer binding to serotonin or in the presence of the negative control 5-HIAA was time-resolved by calculating the measured droplet-averaged fluorescence intensity spectra at different positions along the channel, similar to equilibrium titration measurements. A single exponential fit to each sample concentration yields an observed rate coefficient (*k*_obs_), which is then plotted as a function of serotonin concentration (Figure 2b,c at the bottom).

Unlike the second protocol, which samples time scales in the millisecond range, the third interrogates aptamer–target interactions at slower time scales, between 90 s to 90 min, with tens of seconds resolution. Prior to the measurement, the aptamer and serotonin (or negative control) solutions were mixed in a reaction tube, initiating the reaction, and then immediately loaded into the droplet chip. In the chip, the time-resolved change in ⟨*E*⟩_app._ is measured at a single position along the channel as a function of time.

For both dynamic protocols, a high concentration of the negative control (1 μM 5-HIAA, almost 2 orders of magnitude higher in concentration than the reported 30 nM *K*_D_ for serotonin) is measured as well, showing only minimal change in ⟨*E*⟩_app_, thereby confirming the selectivity of the aptamer and the absence of nonspecific interactions.

For a simple two-state binding mechanism, the observed on-rates, *k*_on, obs._ from both the second and third protocol should yield the same kinetic rate coefficients (given the reaction happens over both time scales) and increase linearly with the concentration of the analyte. If this is not the case, as shown in the examples in Figure 2b,c, a more complex binding mechanism must be considered. This observation highlights, once again, the need for a flexible, multiparametric platform using different measurement protocols. Together, this system allows for a comprehensive characterization of aptamer–analyte interaction kinetics across a broad range of time scales under both equilibrium and nonequilibrium conditions, all within a single microfluidic chip design.

In this work, assays were performed so that, on average, 1000 droplets were collected and averaged to obtain FRET spectra with a high signal-to-noise ratio (SNR). Nevertheless, to showcase the scalability of our approach, we varied the number of averaged droplets per spectrum for aptamer L4 to determine the minimum number needed to retrieve comparable aptamer–target interaction parameters over the three experimental protocols. The results in Figure 3 demonstrate that a single droplet can reliably reproduce the highly averaged data with only minimal deviation, thereby enabling a 1000-fold increase in throughput or 1000-fold decrease in sample consumption. For all different numbers of droplets averaged, the observed K_D_ values remain consistent within error margins (Figure 3a). The observed on-rates on the ms-time scale (Figure 3b) are all within the same order of magnitude (deviation compared to *N* = 1000: 0%, 2%, 18% for *N* = 1, 10, and 20, respectively) and exhibit negligible changes on the min-time scale (Figure 3c). Together, these results demonstrate that our detection approach is compatible with high-throughput screening possibilities based on single droplets, leading to between 100 and 200 potential experiments per second.

**Figure 3:**
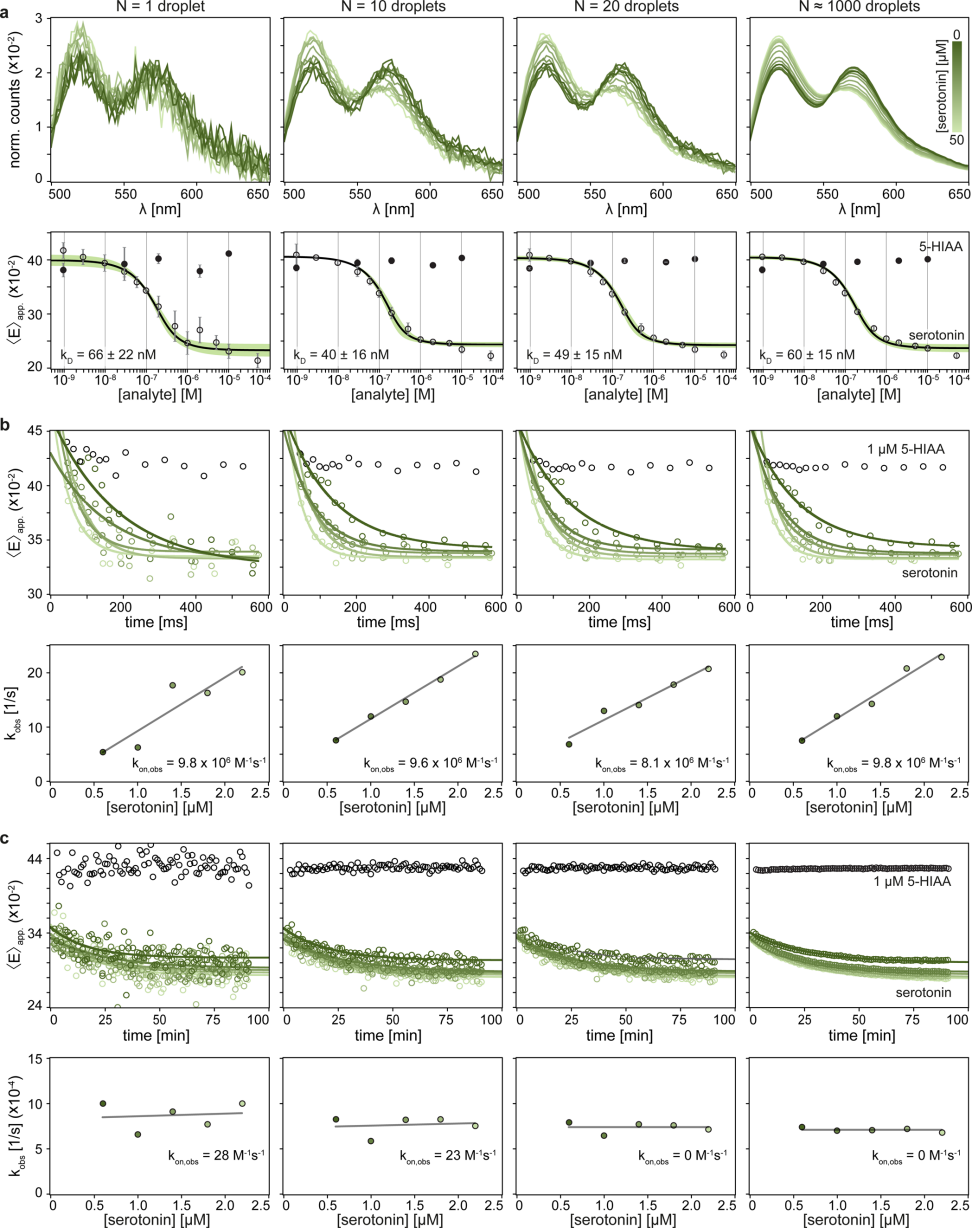
Single-droplet-based screening of aptamer–target interactions. Data for the three measurement protocols as a function of number of droplets averaged in the analysis (*N* = 1, 10, 20, and 1000). (a) Equilibrium titration measurements, (b) dynamic measurements on the ms-time scale, and (c) dynamic measurements on the min-time scale. All data are based on aptamer L4.

### Resolving Different Kinetic Pathways Mediated by Structural Modifications in the Stem Length of the Aptamer

Using the three previously described protocols, we screened a set of serotonin aptamers with varying stem lengths to investigate how structural differences influence the kinetic behavior (Figure 4a). As an initial step, we studied these systems under idealized matrix conditions represented by a buffer (i.e., phosphate-buffered saline, PBS).

**Figure 4:**
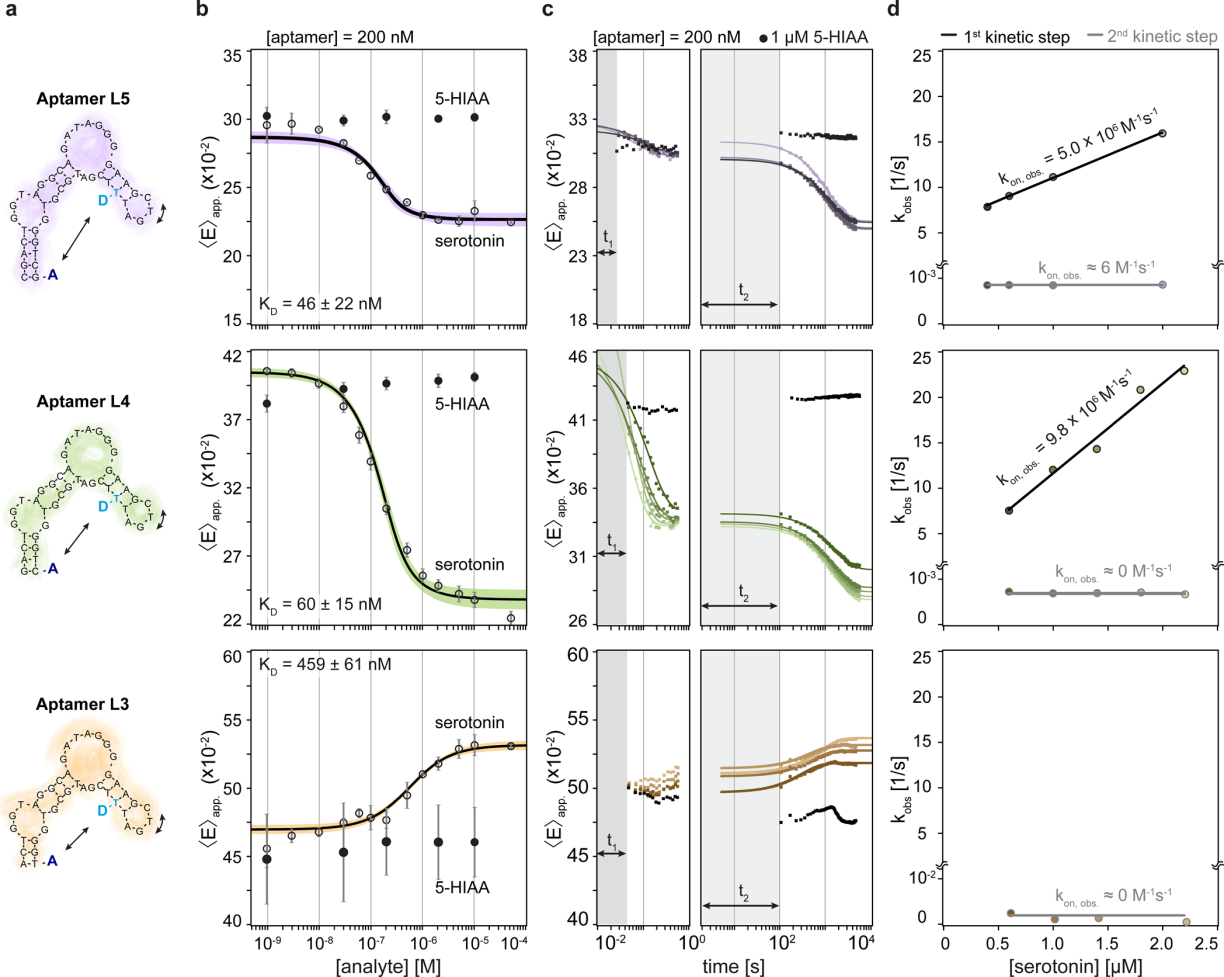
Droplet-based spectroscopy resolves different kinetic pathways mediated by structural modifications in the stem length of the serotonin aptamer. (a) Schematic representation of the three investigated aptamers with varying stem lengths. (b) Equilibrium titration measurements in PBS after previous incubation. The error bar represents the STD for three replicates (different microfluidic chips). For the fit to a sigmoidal binding model, the mean over the three replicates was used and weighted by the variance. The shaded area indicates a confidence interval of 90%. (c) Dynamic measurements for several concentrations of serotonin observed over short (ms) and long (min) time scales. Both time scales were individually fit to exponential models to extract the observed rate constants (*k*_obs_) for each concentration. *t*_1_ and *t*_2_ indicate the dead time related to chip mixing and setting up the droplet experiment, respectively. (d) Plot of *k*_obs_ as a function of the serotonin concentration. The slope of the linear fit represents the on-rate of the aptamer for the specific kinetic state. Note that the on-rate (different for each aptamer) in the first kinetic step is concentration-dependent, while the second kinetic step is concentration-independent for all three aptamers. Also, the different measurements were performed in different microfluidic chips, using different aptamer and serotonin aliquots. For this reason, the *y*-axes do not always align perfectly.

First, equilibrium titration measurements were used to characterize the effect of the stem length on the overall affinity of the aptamer (Figure 4b). For the aptamer sequence with the longest stem, L5, ⟨*E*⟩_app._ decreased with increasing serotonin concentrations, indicating a binding-induced structural rearrangement that increased the distance between the FRET dyes. This conformational change, interpreted as an elongation of the aptamer, aligns with prior experimental and theoretical reports.^17,36,42^ With decreasing stem length (aptamer L4), ⟨*E*⟩_app_ of the free aptamer increases, consistent with the closer dye proximity. Additionally, the increased change in transfer efficiency between both dyes approaches the Förster radius, *R*_0_, enhancing sensitivity to small changes (recall that FRET scales with *r* ^6^). As expected, a further reduction of the stem length (aptamer L3) increases ⟨*E*⟩_app_ of the free aptamer due to the even closer proximity of the FRET dyes. Unlike the other two aptamers, for aptamer L3, an increase in ⟨*E*⟩_app._ was observed upon saturating the aptamer with serotonin, suggesting a different structural rearrangement pathway, where the dyes are brought closer together by the closing of the aptamer–serotonin complex. The control measurements for all three aptamers align well with the baseline (i.e., low serotonin concentrations) of the titration curves and show negligible signal changes with increased sample concentrations.

Notably, *K*_D_ increased as the stem length decreased, indicating a decrease in binding affinity with shorter stem length. While aptamers L5 and L4 have comparable affinities (*K*_D_ = 46 ± 22 nM and *K*_D_ = 60 ± 15 nM for aptamers L5 and L4, respectively), the affinity of aptamer L3 is 10-fold weaker (*K*_D_ = 459 ± 61 nM). To ensure that this trend was not an artifact from the FRET dyes, complementary Thioflavin T (ThT) dye displacement assays^12^ were performed, where the analyte off-competes the aptamer-intercalated dye (see SI and Figure S8). While these measurements do not exactly replicate the absolute binding affinities found using FRET (*K*_D, ThT_ = 139 ± 24 nM, *K*_D, ThT_ = 217 ± 23 nM, and *K*_D, ThT_ = 11.6 ± 1.8 μM for aptamers L5, L4, and L3, respectively), they confirm the general trend that longer aptamer stems result in higher binding affinities. These differences in K_D_ between the two measurements are expected as the ThT assay measures ligand displacement, whereas FRET directly probes the structural switching of the aptamer upon ligand binding.

Next, we investigated the binding kinetics of the different aptamers at fast (ms) and slow (min) time scales (Figure 4c) and analyzed the observed rate coefficients. Regarding kinetics, we observed clear differences between all three aptamers. The aptamer with the longest stem (L5) showed a drop in ⟨*E*⟩_app._ of roughly 25% of the total decay within the first 600 ms (for the highest concentrations of serotonin), followed by a much larger decrease in ⟨*E*⟩_app._ on the longer time scale seemingly decaying independent of the serotonin concentration. Indeed, when analyzed separately, the fast and slow time scales show significantly different apparent association rate constants of *k*_on, obs._ = 5.0 × 10^6^ M^–1^ s^–1^ and *k*_on, obs._ = 6 M^–1^ s^–1^, respectively. These two sets of measurements are thereby reporting on dynamic processes with a well-defined separation of time scales. We attribute the fast kinetic step to the binding of serotonin to the aptamer, with an initial and fast formation of intermediate bound complexes, and the slower kinetic step, independent of the serotonin concentration, to a larger and slower conformational rearrangement to form a more stable aptamer–serotonin complex. The negative control ([5-HIAA] = 1 μM) remained stable over the fast and slow reaction time scales with only minor ⟨*E*⟩_app._ changes, although a slight shift to lower ⟨*E*⟩_app._ values was observed during the fast time scale.

The aptamer with an intermediate stem length (L4) shows similar trends. ⟨*E*⟩_app._ decreases in two steps, first by approximately 65% of the total decay in the first 600 ms, resulting in a nearly 2-fold higher *k*_on, obs._ of 9.8 × 10^6^ M^–1^ s^–1^ compared to aptamer L5. At the slower time scale, aptamer L4 showed a behavior similar to aptamer L5, independent of the serotonin concentration. In accordance with previous observations, the negative control showed a stable signal over time, albeit with a slight shift to lower ⟨*E*⟩_app._ values with respect to the equilibrium titration measurement.

In contrast, the kinetics of the shortest aptamer (L3) differ significantly from the other two, consistent with its different behavior in the equilibrium experiment. On the ms-time scale, the initial ⟨*E*⟩_app._ values were shifted with respect to the equilibrium titration measurement, suggesting that a reaction had already taken place. During the next 500 ms, ⟨*E*⟩_app._ slightly increased; however, the data did not follow the same reaction mechanism as for the other two aptamers. On the longer time scale, the signal change for aptamer L3 was significantly smaller but well aligned with the equilibrium titration measurements. Similar to the other two aptamers, the extracted observed rates at the slower time scales were constant and thereby independent of serotonin concentration (*k*_on, obs._ = 0 M^–1^ s^–1^). The negative control for this aptamer, just like in the titration experiment, also displayed greater signal fluctuations and a shift to higher ⟨*E*⟩_app._ at the first stages of the reaction.

Our experiments revealed a common two-step structural rearrangement across all three aptamers: the initial step (serotonin concentration-dependent) likely involving the binding of serotonin and the formation of intermediate state complexes (fast signal change within less than 1 s), followed by a slower structural rearrangement of the complex (over approximately 60 min) to form the stable aptamer–serotonin complex. Furthermore, the two steps observed in our experiments agree with a recently proposed kinetic pathway model^32^ that is based on four states. The model includes the free aptamer state A + S, the stable complex state AS, and two intermediate states (AS^‡^_1_, AS^‡^_2_) that form two complementary pathways, of which only AS^‡^_2_ shows a transfer efficiency that is significantly different to that of the free aptamer (in our calculation, we fixed the transfer efficiency of AS^‡^_1_ to be the one of the free aptamer and the transfer efficiency of AS^‡^_2_ to be the one of the AS complex). Finally, ⟨*E*⟩_app._ measures the ensemble average of all subpopulation states present at a given time point, weighted by their respective subpopulation fractions. Based on this model, we calculated the population distribution using the specific values observed in our experiments (see SI and Figure S9) for the two longer aptamer sequences. We found that aptamer L5 predominantly interacts with serotonin via populating the AS^‡^_1_ intermediate complex at short times ([AS^‡^_1_] > [AS^‡^_2_]), while aptamer L4 populates both intermediate states to approximately equal amounts ([AS^‡^_1_] ≈ [AS^‡^_2_]). The difference in the intermediate state population fractions between aptamers L5 and L4 (as shown in Figure S9c) accounts for the variation in the decrease of ⟨*E*⟩_app._ observed in the ensemble FRET kinetic experiments (Figure 4c).

Given a sufficient SNR, even a single droplet can be enough to obtain comparable information, as demonstrated in Figure 3. In case the SNR is slightly worse, averaging 10 droplets (Figure S10) is enough to allow for proper readout. This demonstration not only underscores the possible experimental throughput but also highlights the low sample volume requirements of our technique.

### Direct Screening of Aptamer Performance in Complex Biological Medium

A central objective of aptamer engineering and high-throughput screening is to characterize aptamer performance under conditions that closely mimic their intended application. For biosensing in complex biological media such as blood, urine, or saliva, it is critical to ensure that the aptamers maintain functionality in these environments. While characterization of aptamer functionality directly in biological matrices is feasible, it is rarely performed, as it often requires large sample volumes and high concentrations, making such measurements costly and impractical.

Following the initial screening of all three aptamer variations under standard conditions, we selected the two longer aptamer sequences, aptamers L5 and L4, for further investigation in a complex biological medium, due to both their higher affinities and different kinetic pathways. Serum was selected because this serotonin aptamer has previously been shown to function in this media,^18,43,44^ and aptamer activity is highly dependent on ionic conditions.^45^ Using a matrix with established compatibility ensured that our measurements reflected the performance of the platform rather than matrix-induced effects. As a first step, we verified the expected change in the emission profile due to differences in the microenvironment around the dyes of the aptamer when transitioning from pure buffer to 10% human serum (in the absence of serotonin), a phenomenon known as the solvatochromic effect. By recording the full fluorescence spectra, our platform resolves a 1.3 and 1.5 nm shift in the donor dye emission for aptamers L5 and L4, respectively (Figure S11).

Similar to buffer conditions, both aptamers maintain nanomolar affinity and exhibit two-step binding kinetics in human serum: a fast, subsecond concentration-dependent step, followed by a slow, mostly concentration-independent one (Figure 5). Despite the similarities, there are notable differences. For aptamer L5, *K*_D_ decreased from 46 ± 22 nM in PBS to 16 ± 11 nM, despite a reduction of the concentration-dependent *k*_on, obs_, calculated from the fast time-scale measurement by approximately an order of magnitude compared to buffer measurements. Hence, the free energy of the aptamer–serotonin complexes must be further stabilized by the serum environment, compared with the buffer condition. Interestingly, according to the calculated fractions using the proposed model (Figure S9c), the intermediate state AS^‡^_2_ is relatively more populated in human serum in contrast to buffer conditions. This change in the relative population fraction of the two intermediate states indicates that serum affects their relative stability, further supporting the hypothesis that serum conditions affect the free energy of the aptamer–serotonin complexes.

**Figure 5:**
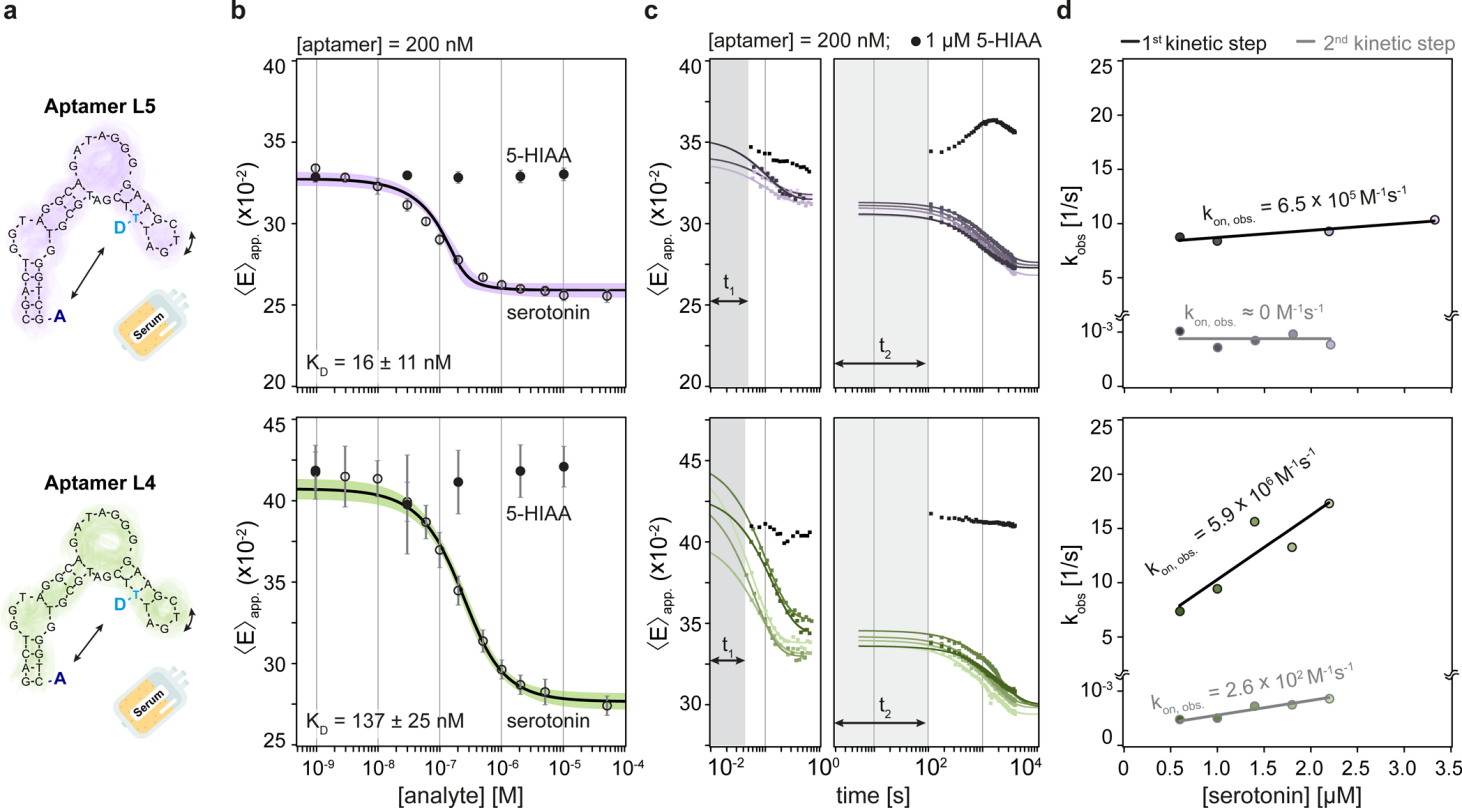
Observation of kinetic pathways in 10% human serum for two variations of the serotonin aptamer. (a) Schematic representation of the investigated aptamer structures in complex media. (b) Equilibrium titration curve after a previous incubation of the aptamer–serotonin mixture. Each data point represents the average of three independent measurements recorded on different microfluidic chips. The error bar depicts the STD over all three replicates. (c) Dynamic measurement over short- and long-time scales for both aptamers. *t*_1_ and *t*_2_ indicate the dead times related to in-chip mixing and setting up the droplet experiment, respectively. (d) Plot of serotonin concentration vs *k*_obs_, as extracted from the exponential fits based on the data presented in panel (c).

For aptamer L4, the dissociation constant increased roughly 2-fold to 137 ± 25 nM (compared to 60 ± 15 nM in PBS), consistent with the 2-fold decrease in the serotonin concentration-dependent apparent association rate coefficient (from 9.8 × 10^6^ M^–1^ s^–1^ in buffer to 5.9 × 10^6^ M^–1^ s^–1^ in human serum). These comparable values suggest that serum conditions marginally affect the stability of the aptamer–serotonin complexes while primarily interfering with the association reaction, likely due to the presence of nonspecific species that interfere with the binding.

Based on this screening, we identified two possible scenarios for FRET-based sensing of serotonin, each optimized for a different aptamer stem length. If high affinity is the only requisite and measurements can be performed over extended periods, aptamer L5 is optimal. However, for rapid testing scenarios, such as point-of-care diagnostics or high-throughput screening of different serotonin-containing samples, aptamer L4 is preferable. This variant enables a concentration-dependent readout extracted in under 1 s, representing an ∼1000-fold reduction in assay time compared to the longer stem aptamer, which requires over 20 min.

## Conclusions

In summary, we presented a scalable approach for multiparametric characterization of aptamers requiring only small sample volumes (pL–nL) and low concentrations. We further demonstrated the reusability of the same chip to quickly screen through different aptamer sequences in buffer conditions and in complex biological media, such as diluted human serum. As a proof-of-concept, we interrogated the influence of the stem length of a serotonin structure-switching aptamer using ensemble-based FRET measurements. By doing so, we identified differences in binding affinities and switching behavior that can be further targeted and tailored to different sensing approaches.

While our proposed technique relies on ensemble-based readout and is therefore unable to retrieve certain information that is specific to single-molecule approaches, we believe it is ideally suited for performing fast initial screenings, especially since only a single droplet is needed per data point readout when the SNR is sufficiently high. Following these screenings, detailed single-molecule studies can then be focused on selecting optimized aptamer–target pairs. In terms of scalability, our approach could be integrated with system-wide automation through the incorporation of programmable motorized stages and more advanced microfluidic designs with additional inlets for higher levels of sample parallelization.^46–49^ This combination would allow for much shorter assay times while also minimizing potential operator errors. Furthermore, despite droplets providing excellent passivation and preventing sample depletion, areas upstream, such as tubing and reservoirs, would still benefit from advanced surface passivation protocols.^50^ Using a more sensitive optical sensor, such as an electron multiplying charge-coupled device (EMCCD), which exhibits not only 10-fold smaller readout noise but also 40-fold higher gains compared to the CMOS chip used here, would allow us to work with at least 100-fold lower sample concentrations. Including more microfluidic building blocks (e.g., dilution modules^47–49,51,52^ and improved mixing structures^41^) could further improve concentration accuracies and measurement dead times within the kinetic assays. Finally, delivering high-throughput screening capabilities across multiple different samples requires the integration of droplet-based sample library generation and droplet tracking, critical bottlenecks in making droplet microfluidic technology a viable alternative to the gold standard of well-plate-based screening.^53^

This droplet-based microfluidic platform bridges key gaps in the current aptamer-based sensor development pipeline by enabling rapid, multiparametric screening of aptamer–target interactions under realistic conditions. We have developed a scalable droplet readout and analysis module with the potential for high-throughput sample screenings. In doing so, this system lays the foundation for the more data-driven, application-specific engineering of next-generation aptamer biosensors.

## Materials and Methods

### Reagents

Microfluidic oil for droplet production, 3M Novec 7500 fluorinated oil containing 2% dSURF (DR-RE-SU-30), was purchased form Fluigent, France, and mixed in a 2:1 v/v ratio with 1,3-bis­(trifluoromethyl)-5-bromobenzene (290157–50g, Merck, Switzerland) to adjust the refractive index of the oil phase to the one of the droplet phase. As a buffer, phosphate-buffered saline (PBS, pH 7.4, 10010, Thermo Fisher Scientific, Switzerland) was used for all experiments. Serotonin (H9523), 5-hydroxyindole-3-acetic acid (5-HIAA, H8876), and anonymized human serum were all purchased from Merck, Switzerland.

### Aptamer Sequences

All aptamer sequences were purchased from Microsynth, Switzerland, and received at a concentration of 100 μM in deionized water. The stock solution was diluted to 10 μM using PBS and aliquoted in volumes between 20 and 30 μL. The exact sequences are listed below from 5′ to 3′:

Aptamer L5, donor-only: CGA CTG GTA GGC AGA TAG GGG AAG CTG AT­(FAMdT) TCG ATG CGT GG TCG

Aptamer L5, FRET: CGA CTG GTA GGC AGA TAG GGG AAG CTG AT­(FAMdT) TCG ATG CGT GG TCG-(TAMRA)

Aptamer L4, FRET: GAC TGG TAG GCA GAT AGG GGA AGC TGA T­(FAMdT)­T CGA TGC GTG GGT C-(TAMRA)

Aptamer L3, FRET: ACT GGT AGG CAG ATA GGG GAA GCT GAT (FAMdT)­TC GAT GCG TGG GT-(TAMRA)

### Measurement Conditions

All droplet-based experiments (if not otherwise indicated) were recorded using the parameters stated below. Any further information required is given directly in the respective paragraphs.

Chip preparation: All microfluidic chips were bleached with a high-power LED (λ = 480 nm, power density 2.65 mW/mm^2^, M470L2, Thorlabs, USA) for several hours to reduce autofluorescence contributions from the PDMS and the used glass slides prior to being used for any experiment. Moreover, to avoid depletion, all sample reservoirs and inlet tubing, as well as the chip itself, were passivated with 100 μM serotonin, rinsed twice with buffer, and then dried with N_2_.

LASER settings: The LASER was operated with a 487 nm diode at a current of 95 mA.

Camera settings: 750 μs exposure time, 500 fps, 6000 recorded frames, 30-fold gain for the HSI camera, and 150 μs exposure time, 10 fps, 30 recorded frames for the big field of view camera.

Pressure settings: The control layer was pressurized with 2000 mbar, for the flow layer (i.e., sample and oil phase) the pressure was set between 100 and 500 mbar depending on the required droplet speed. This corresponds to a droplet production rate between 100 and 200 Hz.

### Model for Equilibrium Titration Measurements

In the equilibrium titration measurements, we observe the change in the apparent FRET efficiency ⟨*E*⟩_app._ as a function of the serotonin concentration, effectively reporting on the fraction of aptamer bound to serotonin θ_AS_:

$$\theta_{\mathrm{AS}}=\frac{\left[\mathrm{AS}\right]}{\left[\mathrm{AS}]+[A\right]}$$

where [AS] is the concentration of the aptamer bound to serotonin, and [A] is the concentration of the free aptamer. In combination with the formula for the dissociation constant:

$$K_{D}=\frac{\left[A][S\right]}{\left[\mathrm{AS}\right]}$$

with [S] being the concentration of free serotonin. Using the binding isotherm for a nonsimplified system and the notation [A]_tot_ = [A] + [AS] and [S]_tot_ = [S] + [AS], we can rewrite θ_AS_ as

$$\theta_{\mathrm{AS}}=\frac{([A{]}_{\mathrm{tot}}+K_{D}+[S{]}_{\mathrm{tot}})-\sqrt{([A{]}_{\mathrm{tot}}+K_{D}+[S{]}_{\mathrm{tot}}{)}^{2}-4[A{]}_{\mathrm{tot}}[S{]}_{\mathrm{tot}}}}{2[A{]}_{\mathrm{tot}}}$$

We refer the interested reader to the appendix of Copeland^54^ for a full derivation.

Since these experiments are performed in a regime where the aptamer concentration is [A] ≈ *K*_D_, this formula cannot be further simplified without making any assumptions.

Finally, since the experiments are based on ensemble measurements, the observed transfer efficiency signal is ⟨*E*⟩_app._ = θ_AS_⟨*E*⟩_AS_ + θ_A_⟨*E*⟩_A_, where ⟨*E*⟩_A_ and ⟨*E*⟩_AS_ are the FRET efficiency of the free and bound aptamer respectively, and θ_A_ is the fraction of free aptamer (θ_A_ = 1 – θ_AS_). Then, ⟨*E*⟩_app._ can be written as

$$\langle E\rangle_{\mathrm{app}}=\Delta \times \theta_{\mathrm{AS}}+c$$

where *c* = ⟨*E*⟩_A_, and the scaling factor Δ = ⟨*E*⟩_AS_ – ⟨*E*⟩_A_.

For all measurements, [A]_tot_ was fixed to 200 nM (FRET measurements) or 400 nM (ThT assay), and [S]_tot_ was used up to 50 μM, while all other variables were kept as free fitting parameters.

### Fit Model for Dynamic Measurements

All data recorded in dynamic measurements were fitted with an exponential function ⟨*E*⟩_app._ = *Ae* ^–*kt*^ + *B*, with *A*, *B* being the amplitude and offset of the change in transfer efficiency, respectively, while *k* is the value of the observed rate of change in transfer efficiency.

## Supplementary Material



## Data Availability

The main data supporting the results of this study are available within the paper and its Supporting Information. The raw and analyzed data sets generated during the study are too large to be publicly shared yet are available for research purposes from the corresponding authors upon request. Requests will be fulfilled within 10 weeks.

## Notes

Detailed description of the optical setup and its calibration; fabrication and integration of the microfluidic chips; data read-out principle; sheath flow and several measurements regarding sample parameters and external influences, such as focus position; complementary ThT assay data and a 4-state model regarding the binding of serotonin to the aptamer; data evaluation based on only 10 droplets averaged and two plots regarding the solvatochromic effect (PDF)

## References

[1] Moraldo C., Vuille-dit-Bille E., Shkodra B., Kloter T., Nakatsuka N. (2022). Aptamer-modified
biosensors to visualize neurotransmitter flux. J. Neurosci. Methods.

[2] Yang K. (2023). A functional
group-guided approach to aptamers for small molecules. Science.

[3] Zhou J., Rossi J. (2017). Aptamers as targeted therapeutics: Current potential and challenges. Nat. Rev. Drug Discovery.

[4] Yang L. F., Ling M., Kacherovsky N., Pun S. H. (2023). Aptamers 101: Aptamer
discovery and in vitro applications in biosensors and separations. Chem. Sci..

[5] Yang K. A. (2017). High-affinity nucleic-acid-based receptors for steroids. ACS Chem. Biol..

[6] Baker M. (2015). Reproducibility
crisis: Blame it on the antibodies. Nature.

[7] Frutiger A. (2021). Nonspecific binding
- Fundamental concepts and consequences for biosensing
applications. Chem. Rev..

[8] Nakatsuka N. (2018). Aptamer-field-effect
transistors overcome debye length limitations
for small-molecule sensing. Science.

[9] Feagin T. A., Maganzini N., Soh H. T. (2018). Strategies for creating structure-switching
aptamers. ACS Sens..

[10] Yoshikawa A. M. (2023). A massively parallel
screening platform for converting aptamers into
molecular switches. Nat. Commun..

[11] Wu Y. (2023). Using spectroscopy to guide the adaptation of aptamers
into electrochemical
aptamer-based sensors. Bioconjugate Chem..

[12] Yang K. (2024). Exploring the landscape
of aptamers: From cross-reactive to selective
to specific, high-affinity receptors for cocaine. JACS Au.

[13] Li S. (2023). Implantable
hydrogel-protective DNA aptamer-based sensor supports
accurate, continuous electrochemical analysis of drugs at multiple
sites in living rats. ACS Nano.

[14] Downs A. M., Plaxco K. W. (2022). Real-time, in vivo
molecular monitoring using electrochemical
aptamer based sensors: Opportunities and challenges. ACS Sens..

[15] Reynoso M. (2024). 3D-printed, aptamer-based microneedle sensor arrays
using magnetic
placement on live rats for pharmacokinetic measurements in interstitial
fluid. Biosens. Bioelectron..

[16] Stuber A. (2024). Interfacing aptamer-modified nanopipettes with neuronal
media and
ex vivo brain tissue. ACS Meas. Sci. Au.

[17] Stuber A. (2023). Aptamer conformational
dynamics modulate neurotransmitter sensing
in nanopores. ACS Nano.

[18] Nakatsuka N. (2021). Sensing serotonin secreted
from human serotonergic neurons using
aptamer-modified nanopipettes. Mol. Psychiatry.

[19] Nakatsuka N. (2021). Aptamer conformational
change enables serotonin biosensing with nanopipettes. Anal. Chem..

[20] Alkhamis O. (2025). Exploring the relationship between aptamer
binding thermodynamics,
affinity, and specificity. Nucleic Acids Res..

[21] Mayer, G. ; Menger, M. M. Nucleic acid aptamers: Selection, characterization, and application. Methods in Molecular Biology, 2nd ed.; Humana: New York, 2023, 2570.

[22] Yang Y. (1996). Structural basis of ligand discrimination by two related
RNA aptamers
resolved by NMR spectroscopy. Science.

[23] Osypova A. (2015). Sensor based on aptamer
folding to detect low-molecular weight analytes. Anal. Chem..

[24] Jensen H., Østergaard J. (2010). Flow induced dispersion analysis quantifies noncovalent
interactions in nanoliter samples. J. Am. Chem.
Soc..

[25] Otzen D. E., Buell A. K., Jensen H. (2021). Microfluidics
and the quantification
of biomolecular interactions. Curr. Opin. Struct.
Biol..

[26] Jeng S. C. Y. (2021). Fluorogenic aptamers resolve the flexibility of RNA
junctions using orientation-dependent FRET. RNA.

[27] Zimmer K. J. L., Johnson R. E., Little H., Duhamel J., Manderville R. A. (2025). Harnessing
a fluorescent nucleobase surrogate for supramolecular FRET-aptamer
detection and target-site mapping. ACS Sens..

[28] Jepsen M. D. E. (2018). Development of a genetically
encodable FRET system
using fluorescent RNA aptamers. Nat. Commun..

[29] Filius M., Fasching L., Wee R., van Rwei A. Y., Joo C. (2025). Decoding aptamer-protein
binding kinetics for continuous biosensing using single-molecule techniques. Sci. Adv..

[30] Severins I. (2024). Single-molecule structural
and kinetic studies across sequence space. Science.

[31] Zosel F., Holla A., Schuler B. (2022). Labeling of
proteins for single-molecule
fluorescence spectroscopy. Methods Mol. Biol..

[32] Perez-Gonzalez C., Lafontaine D. A., Penedo J. C. (2016). Fluorescence-based strategies to
investigate the structure and dynamics of aptamer-ligand complexes. Front. Chem..

[33] Lee Y. (2024). Carbon-nanotube field-effect
transistors for resolving single-molecule
aptamer–ligand binding kinetics. Nat.
Nanotechnol..

[34] Ren R. (2017). Nanopore
extended field-effect transistor for selective single-molecule
biosensing. Nat. Commun..

[35] Squires T. M., Messinger R. J., Manalis S. R. (2008). Making it stick: Convection, reaction
and diffusion in surface-based biosensors. Nat.
Biotechnol..

[36] Douaki A. (2023). Theoretical analysis
of divalent cation effects on aptamer recognition
of neurotransmitter targets. Chem. Commun..

[37] Shaver A. (2022). Optimization of vancomycin
aptamer sequence length increases the
sensitivity of electrochemical, aptamer-based sensors in vivo. ACS Sens..

[38] Kaiyum Y. A. (2024). Ligand-induced folding in a dopamine-binding
DNA aptamer. ChemBioChem.

[39] Sulliger M., Ortega Arroyo J., Quidant R. (2025). Hyperspectral imaging for high throughput
optical spectroscopy of pL droplets. Anal. Chem..

[40] Unger M. A., Chou H.-P., Thorsen T., Scherer A., Quake S. R. (2000). Monolithic
microfabricated valves and pumps by multilayer soft lithography. Science.

[41] Yang T. (2023). Rapid droplet-based mixing for single-molecule spectroscopy. Nat. Methods.

[42] Stuber A., Nakatsuka N. (2024). Aptamer renaissance for neurochemical
biosensing. ACS Nano.

[43] Tran B. N. (2024). Minimizing false positives
in gold nanoparticle-aptamer biosensors
for enhanced serotonin detection. ACS Appl.
Nano Mater..

[44] Song G. (2023). Light-up aptameric sensor of serotonin for point-of-care use. Anal. Chem..

[45] Nakatsuka N., Abendroth J. M., Yang K. A., Andrews A. M. (2021). Divalent
cation
dependence enhances dopamine aptamer biosensing. ACS Appl. Mater. Interfaces.

[46] Wu J., Hwang Y. H., Yadavali S., Lee D., Issadore D. A. (2024). Micro-patterning
wettability in very large scale microfluidic integrated chips for
double emulsion generation. Adv. Funct. Mater..

[47] Ganguly R., Lee C. S. (2024). Poisson-independent approach to precision nucleic acid
quantification in microdroplets. ACS Appl. Bio
Mater..

[48] Shi Q. (2023). Fluorescence-coded
logarithmic-dilution digital droplet PCR for ultrawide-dynamic-range
nucleic acid quantification. Biosens. Bioelectron..

[49] Wang X., Liu Z., Pang Y. (2017). Concentration
gradient generation methods based on
microfluidic systems. RSC Adv..

[50] Belling J. N. (2020). Lipid-bicelle-coated
microfluidics for intracellular delivery with
reduced fouling. ACS Appl. Mater. Interfaces.

[51] Lee K. (2009). Generalized serial dilution
module for monotonic and arbitrary microfluidic
gradient generators. Lab Chip.

[52] Wang Y. H., Sun Y. S. (2023). Use microfluidics
to create microdroplets for culturing
and investigating algal cells in a high-throughput manner. Microfluid. Nanofluid..

[53] Payne E. M., Holland-Moritz D. A., Sun S., Kennedy R. T. (2020). High-throughput
screening by droplet microfluidics: Perspective into key challenges
and future prospects. Lab Chip.

[54] Copeland, R. A. Evaluation of Enzyme Inhibitors in Drug Discovery: A Guide for Medicinal Chemists and Pharmacologists, 2nd ed.; Wiley: Hoboken, NJ, 2013. 16350889

