# Supplementary material for: Scalable Multiparametric Characterization of Aptamer–Target Interactions
Source: ACS Nano. 2026 Jan 8;20(2):2387–98. doi: 10.1021/acsnano.5c19596 (PMC12825375; doi:10.1021/acsnano.5c19596)
Supplement: Supplementary file 1 [file nn5c19596_si_001.pdf]

SUPPORTING INFORMATION

Scalable Multiparametric Characterization of Aptamer-Target Interactions

Marc Sulliger[1], Matthew Peters[1], Andrea Sottini[1], Annina Stuber[2,3], Kyungae Yang[4], Nako Nakatsuka[3,*], Jaime Ortega Arroyo[1,*], Romain Quidant[1]

[1] Nanophotonic Systems Laboratory, Department of Mechanical and Process Engineering, ETH Zurich, 8092 Zurich, Switzerland.

[2] Laboratory of Biosensors and Bioelectronics, Department of Information Technology and Electrical Engineering, ETH Zurich, 8092 Zurich, Switzerland

[3] Laboratory of Chemical Nanotechnology, Neuro-X Institute, EPFL, 1202 Genève, Switzerland

[4] Department of Medicine, Columbia University Irving Medical Center, New York, 10032, NY, United States

Correspondence to:

* jarroyo@ethz.ch, nako.nakatsuka@epfl.ch

Table of Contents:

Supporting Information and Figures……pages S2 – S11

References………………………………page S11

SUPPORTING INFORMATION AND FIGURES

Microscope

The custom-built microscope (Fig. S1) follows a flexible and modular approach combining three illumination sources with three detection modules to form three independent optical paths: a fluorescence based HSI path, a big FoV path and a confocal path.

The core of the setup, the HSI path, is built in a reflection configuration. For fluorescence excitation a free space LASER with a wavelength of 487 nm (P = 69 mW, 40A-45a-48A-64A-11-NT-CF; S/N 909604, Integrated Optics, Lithuania) is used. The beam first passes through a f = 300 mm cylindrical lens (CL1, LJ1558RM, Thorlabs) that acts as a virtual slit. Afterwards it passes through a dichroic mirror (DM1, DMLP425, Thorlabs, USA) and a 1:1 relay-imaging system formed by two f = 125 mm achromatic doublet lenses (L1 and L2, AC254-125-A, Thorlabs). From there, a dichroic mirror (DM2, long pass 490 nm, DMLP490L, Thorlabs) couples the light to the rest of the optical system. Light reflected off from the dichroic mirror is then focused onto the sample using a 4x, 0.2 NA objective (Obj., TL4S-SAP, Thorlabs). The sample is positioned on a custom-built sample holder placed on top of xyz-piezo translation stage (nanoCube, Physik Instrumente GmbH, Germany) mounted on top of a manual xyz-translations stage with a travel range of 25 mm x 25 mm x 12.5 mm. The light emitted from the sample is collected by the same objective, passes through the previously mentioned dichroic mirror and a long pass emission filter (F1, FELH500, Thorlabs) and gets imaged onto a slit (slit, width = 50 µm, VA100/M, Thorlabs) by a f = 150 mm achromatic doublet lens (L3, AC508-150A, Thorlabs) to produce a quasi 1D image. A second f = 150 mm achromatic doublet lens (L4, AC508-150A, Thorlabs) relay images the BFP of the objective onto two subsequent but counter-rotated Amici prisms (117240, Equascience, France) to spectrally disperse the light perpendicular to orientation of the slit. To allow for a multichannel readout a 90:10 (R:T) beam splitter (BS1, BSX16, Thorlabs) was introduced before the Amici prisms. Finally, the light reflected from the beamsplitter and dispersed by the prisms was imaged onto a CMOS camera (HSI cam, GS3-U3-23S6H, Sony IMY174 Sensor, 1200 x 1920 pixel, 5.86 µm x 5.86 µm pixel size, Point Grey, FLIR Systems, USA) by a f = 200 mm achromatic doublet lens (L5, AC508-200-A, Thorlabs). This channel provided an effective 4x magnification (i.e. 1.49 µm/pixel, equal to the spatial resolution) and an average spectral dispersion of 0.782 nm / pixel between 504 - 613 nm.

The big FoV path corresponds to a spatially incoherent digital holographic optical system which is based on a common-path microscope operating in reflection with all optical elements arranged in a 4f configuration. For the illumination, a 415 nm fiber coupled light emitting diode (M415F3, Thorlabs) is connected to a 600 µm multimode fiber (M29L02, Thorlabs). The light gets out-coupled from the fiber by a f = 6.2 mm aspheric lens (AL1, C171TMD-A, Thorlabs) reflects off a dichroic mirror (DM1, DMLP425, Thorlabs) and then follows the same path as the fluorescence excitation. In the imaging arm, light from the sample is again collected by the same objective and subsequently imaged onto a CMOS camera (big FoV cam, a2A4504-18umPRO – Basler ace 2, 4504 x 4504 pixel, 2.74 µm x 2.74 µm pixel size, Basler AG, Germany) upon reflecting off a 50:50 beamsplitter (BS2, BSW27, Thorlabs) that separates the illumination arm for the imaging arm and passing a short pass optical filter (F2, FESH0450, Thorlabs).

Finally, the confocal detection path uses a transmission illumination based on a 740 nm fiber coupled light emitting diode (M740F2, Thorlabs, USA) was connected to a 550 µm multimode fiber (M37L02, Thorlabs). The output of the fiber was imaged onto the sample by a telescope comprising a f = 11 mm aspheric lens (AL2, C397TMD-A, Thorlabs) and a f = 40 mm achromatic doublet lens (L6, AC254-040-A, Thorlabs) arranged in a 4f configuration. The transmitted light follows the HSI path up to the point of the 90:10 beamsplitter, where the reflected light is blocked by an optical filter (F3, FESH0700, Thorlabs) and the transmitted light focused using a f = 150 mm achromatic lens (L7, LA1433-A, Thorlabs) onto an APD (APD, APD120A2/M, Thorlabs) with a 50 µm pinhole (I1, P50K, Thorlabs) mounted right in front of it. All optical elements were arranged in a 4f configuration with respect to the slit, leading to an effective 3x magnification.

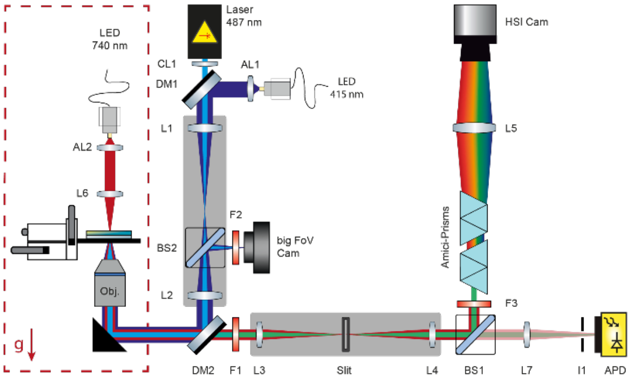

Fig. S1: Schematic representation of the optofluidic platform. The optofluidic setup is equipped with three illumination modules (dark red = transmission illumination, purple = big FoV reflection illumination, light blue = fluorescence excitation) and three detection channels (HSI, big FoV and confocal APD). Within the dashed red box, g indicates the direction of gravity.

Calibration of HSI camera (mapping pixels to wavelength)

The HSI path of the setup as described above is based on a simple off-the-shelf camera which provides images in a pixel reference system. Thereby, the y-axis corresponds to spatial information whereas the x-axis encodes spectral information. To read-out the latter, a coordinate transformation from pixels to wavelength is required. This is achieved by a one-time calibration using a total of nine filters (FF02-472/30-25, Semrock, USA, and MF497-16, FL532-10, FB570-10, FLH635-10, FB650-10, FB700-10, FL740-10, FGB67, Thorlabs) with sharp absorption peaks and a broadband light source (MINTF4, Thorlabs) that temporarily replaces the 740 nm LED of the transmission path. In short, the spectral filters (mounted in front of the light source before the sample stage) are imaged along the HSI path (2000 frames recorded over 40 s, temporally averaged) and compared against 100 temporally averaged scans of a reference spectrometer (USB4000, Ocean Optics, Ocean Insight, USA). The wavelength observed by the spectrometer is then mapped using a 3rd order polynomial to the pixel detecting the intensity maximum in the HSI. The fit is performed for each spatial pixel of the HSI to also account for any smile aberrations of the HSI system. Finally, the resulting fit coefficients are then used to perform the coordinate transformation of the wavelength axis.

Spectral window and data binning

The described implementation of the setup reads spectral information between 500-700 nm. The lower and upper limit are given by the color filters in the HSI path (long pass 500 nm and short pass 700 nm). Regarding data binning, we currently work with a spectral resolution of 2 nm by pixel binning along the spectral axis. Even though the spectral dispersion of our system lays below 1 nm per pixel, a resolution below 2 nm is not required since we already fulfil the Nyquist criteria for the FRET signal.

Fabrication of Molds and Microfluidic Chips

Microfluidic chips were fabricated by means of standard soft lithography. First, direct LASER writing (µMLA, Heidelberg Instruments, Germany) on a 4 inch silicon wafer (WSM4052525XB1314SNN1, MicroChemicals GmbH, Germany) was used to fabricate individual molds for control and flow layer (Fig. S2a). For the control layer mold, a single exposure of 20 µm SU8 GM1060 negative photo resist (Gersteltec Engineering Solutions, Switzerland) was performed. For the flow layer mold two subsequent structuring steps with different photoresist were required to simultaneously allow for semi-rounded (valve areas) and squared (all other areas) channel cross sections. For this reason, the wafer was first coated with 80 µm SU8 GM 1070 negative photo resist, exposed and developed. Then, in a second step, the same wafer was coated with 50 µm IPS 6090 positive photoresist (Micro-Chemicals GmbH). To ensure a uniform resist layer in between pre-existing structures, the wafer was afterwards placed in a gentle vacuum (100 mbar) to remove bubbles and exposed to a mild acetone environment for a few minutes allowing the resit to slightly liquify which smoothed out irregularities in layer thickness. Next, the design was aligned with the previously fabricated SU8 structures, exposed with the same LASER writer, and developed as well. A subsequent baking step then causes the AZ structures to slightly melt and reshape into semi-rounded cross sections (with an arc height of 80 µm) whilst the SU8 photo resist underwent a glass transition but maintains the squared channel profile. Finally, both molds were silanized with chlorothrimethylsilane (92360, Sigma Aldrich, Switzerland) at low pressure to improve longevity.

For the actual droplet chip fabrication, the structures from the two molds were transferred to PDMS (Sylgard 184, Dow Chemical, USA) using a 10:1 w/w polymer to curing agent ratio. For the control layer, a small amount of PDMS was drop-cast into the center of the mold and then spin coated at 2000 rpm resulting in a layer thickness of approximately 40 µm. To produce the flow layer, the PDMS was simply drop-cast onto the respective mold to a height of about 5 mm. Before the molds were baked in a convection oven at 80 °C for one hour, they were placed under vacuum to degas until no more bubbles are visible. Once cured, the PDMS of the flow layer mold was peeled off the substrate (Fig. S2b), cut into individual chips and inlet and outlet holes were punched. These PDMS pieces and the control layer (still attached to the mold) were then exposed to an oxygen plasma (10s, 300 W, 8 sccm, Atto Low Pressure Plasm System, Diener Electronics, Germany) before manual alignment under a stereo macroscope (Z16 APO, Leica, Germany). Subsequently, for a stronger bond between the two layers, the aligned chips were baked again for 15 min at 80 °C in the oven. Afterwards, the chips were separated from the mold and the remaining inlets of the control layer were punched. Finally, to finish the assembly, the prepared PDMS chips were bonded (Fig. S2c) to a microscope glass slide (50 x 24 mm, 170 ± 10 µm, 41014551, Karl Hecht, Switzerland) again using the same plasma and baking conditions as discussed before.

Integration and Interfacing of microfluidic chips with optofluidic setup

The sample inlets of the microfluidic chips were connected to Tygon® tubing (øin = 0.508 mm, øout = 1.524 mm, Saint-Gobain, France) via a bent metal pin (Tube AISI 304, 0.65 / 0.35 x 17.5 mm, Unimed SA, Switzerland). Tubings feeding the flow layer inlets were either connected to 1.5 mL Eppendorf tubes interfaced with a P-Cap (P-CAP2-HP, Fluigent, France) or, for smaller volumes, dispensing tips (LL ½" ID 0.34 mm, GONANO Dosiertechnik GmbH, Austria) with a Male Luer Lock to Barb Adapter (Darwin

Microfluidics, France). These reservoirs were connected to pressure controllers (LU-FEZ-2000, Fluigent) via 4 mm x 2.5 mm polyurethane tubing (917-2407, RS Pro, GB). Said controllers were operated using LineUP Link module (LU-LNK-002, Fluigent) and A-i-O 2020 software (Fluigent). Inlets interfacing with the control layer used the same metal pins and liquid filled Tygon® tubing as described above but connected to a custom-built and programmatically controlled electronic valve unit.

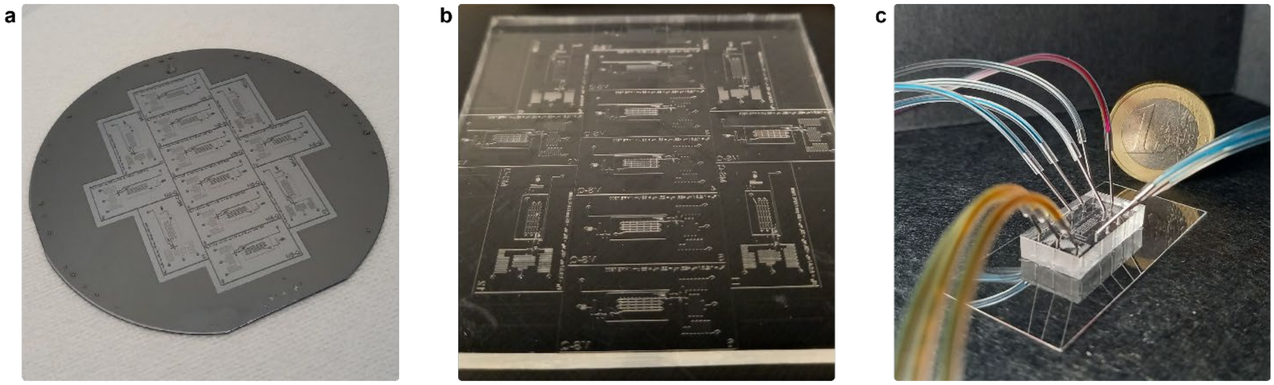

Fig. S2: Microfluidic mold, PDMS and assembled chip. (a) Photograph of flow layer mold hosting a total of 12 chips with identical designs. (b) Cured PDMS slab with reproduced designs based on the mold shown in (a). (c) Image of the assembled microfluidic chip compared to a 1 Euro coin. Blue inlets connect to the control layer. Sample and oil inlets are indicated in orange and purple, respectively. Transparent tubing connects to outlets.

Read-out Principle for Spectral Data

Spectral data analysis is based on the raw HSI images which are in a first step mapped to the wavelength reference system as previously described. Once this is done, we refer to the HSI images as HSI stack (Fig. S3a). Next, the entire HSI stack is temporally averaged resulting in a convolution of droplet and LASER position, effectively reporting on the extend of the area irradiated by the LASER. A line profil through the irradiation maximum defines the beam profile and is then used to set the ROI. Thereby, the ROI is always kept in the centre of the illuminated area with an extent that is narrower than the FWHM of the beam profile itself (Fig. S3b). Once the ROI is defined – we typically use a maximum size of 60 spatial pixels – we go back to the individual HSI images and read out the centre position of each droplet in every frame. Based on the located droplet centre position, each frame is assigned to one of three categories: (i) frames containing droplets overlapping with ROI, (ii) frames containing droplets not overlapping with ROI, and (iii) frames not containing any droplets. The last category is temporally averaged to a single frame which is subsequently subtracted from all frames containing droplets within the ROI (i.e. first category), thereby accounting for dark counts, auto-fluorescence and matrix effects. Finally, using the known droplet centre position for the frames in the first category, a fixed number of spectra are read out around the centre of the droplet (typically 30 spectra) and averaged to a single spectrum. These individual droplet spectra are shown in Fig. S3c. To increase the SNR, all of the individual droplet spectra are then averaged to a single spectrum, which is later on processed to extract information about the investigated system.

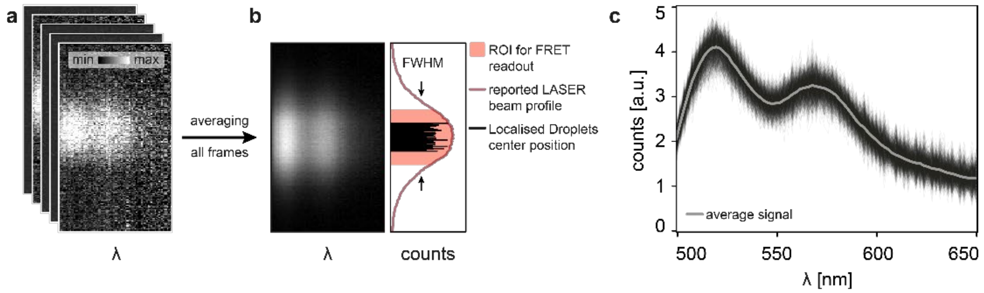

Fig. S3: Principle of FRET signal read-out. (a) Representative stack of raw but spectrally mapped HSI images with visible aptamer signal. (b) Average over all recorded frames (left) reporting on the convolution of the LASER beam and the droplet signal. The panel on the right shows a line profile along the special axis of the LASER signal, allowing to define the illumination centre which is then used to define a ROI for the droplet readout. All droplet positions are localised along the special axis and, given that they overlap with the ROI, used to read out 30 spectra in the centre of said droplets. (c) Spectral read-out of all droplets within the ROI for a given data set (i.e. single droplet spectra) in black and average over all spectra in the data set (grey).

Measurement Parameter

Before performing any kinetic experiments, the measurement parameters for the given aptamer samples have been investigated. This involves the exposure time, the droplet speed and the concentration response of the investigated aptamers.

For the first parameter, the exposure time, droplets loaded with 200 nM of aptamer L5 were continuously produced with a speed of v = 52 ± 1 mm/s and observed at a fixed position whilst adjusting the exposure time from 100 µs up to 3.0 ms. The recorded data has then been analysed in terms of donor peak amplitude as well as based on the apparent transfer efficacy $\langle E \rangle_{app.}$ and is shown in Fig. S4a. Thereby, the donor peak amplitude first increases linearly up to roughly 1.1 seconds, before deviating from the initially linear trend which indicates that blurring is starting to occur. The apparent transfer efficiency on the other hand, stays approximately constant even when blurring starts to affect the measurement.

Next, using a fixed exposure time of 750 µs and a sample concentration of 200 nM, we produced droplets with various velocities and observed the signal at a fixed position. Fig. S4b depicts the same two metrics that were used before and nicely shows that the transfer efficiency stays constant for all droplet velocities, whilst the doner signal starts decreasing (and the STD quickly increases) after reaching a velocity of approximately 75 mm/s. This matches very well with the data observed for the exposure time, which suggests that there should not occur any blurring up to a droplet velocity of 76 mm/s when reducing the exposure time to 750 µs.

After this, we measured a concentration vs. signal calibration curve. To do so, the measurement was performed sequentially for all three aptamers and in order of increasing sample concentration. The droplet speed was always kept below the critical value and all samples were once again observed at the same point. As presented in Fig. S4c, the signal shows a linear increase when considering the donor amplitude and a rather stable signal for the $\langle E \rangle_{app.}$ over all concentrations.

Based on these three experiments, we fixed the exposure time for all experiments in this work to 750 µs (which, therefore, sets the temporal resolution) and the aptamer concentration to 200 nM. Furthermore, we limit ourselves to droplet velocities below 80 mm/s, even though it would not affect any of the read-outs based on transfer efficiency. Using these parameters, Fig. S4d further depicts the time evolution of the $\langle E \rangle_{app.}$ for regular measurements of aptamer L5 over almost 6 h. Finally, Fig. S4e shows concentration calibration curves for all three aptamer variations.

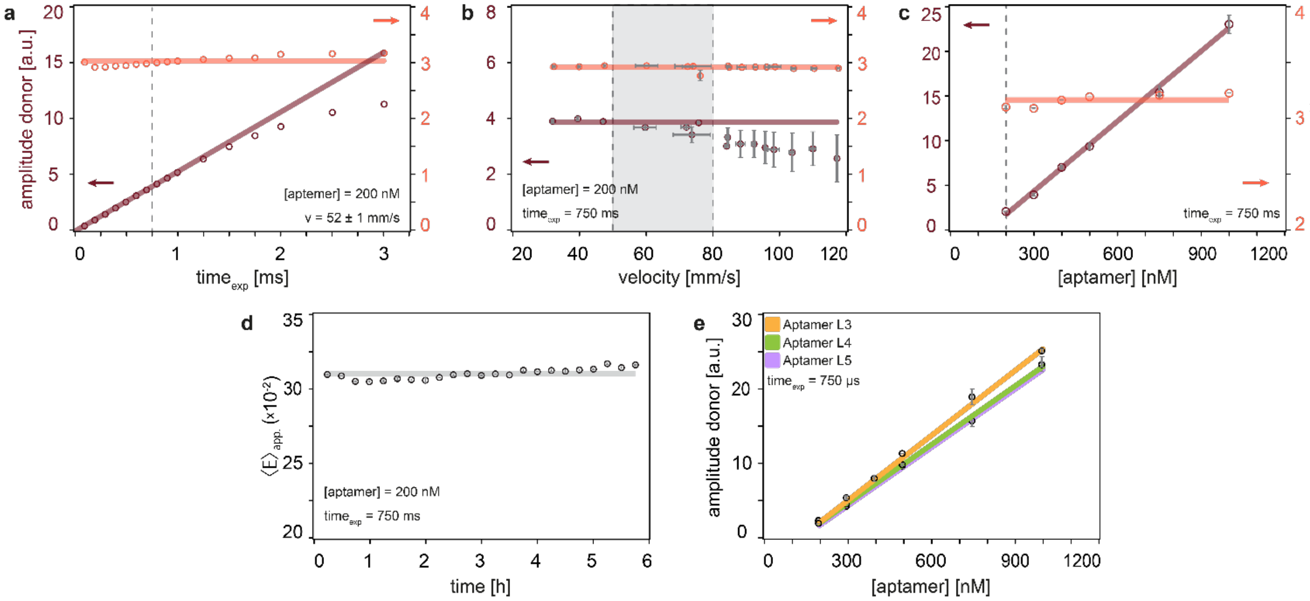

Fig. S4: Relevant measurement parameters. (a) Determination of max. exposure time for a fixed droplet velocity. (b) Possible droplet speeds for a fixed exposure time. (c) Validation of linear and constant signal evolution for donor amplitude and apparent FRET efficacy, respectively, for increasing aptamer concentration. (d) Validation of FRET signal stability over time. (e) Calibration of aptamer signal for all three stem loop configurations. Gray dashed lines in (a) and (c) and the grey shaded area in (b) indicate the used parameters for the experiments documented in this work. All error bars represent STD over three measurements performed in the same microfluidic chip in close temporal proximity. If not noted otherwise, aptamer L5 was used for these measurements.

Focus Influence

For the dynamic measurements, the FRET signal of the sample had to be observed at different time points which translates to different positions along the microfluidic channel. This has been achieved by repositioning the microfluidic chip using the translation stages of the sample holder. Such lateral movements always come at the risk of slightly changing the focus position. Fig. S5 investigated the exact influence of said position changes. Thereby it was found that a relative defocusing causes an increase of the amplitude of the FRET signal (Fig. S5b) and hence improves the SNR. However, upon normalization, the FRET signals for all focus position collapse. Therefore, the read out of observed transfer efficacies from normalized FRET signals is (apart from a slight change in SNR) not affected by the focus position.

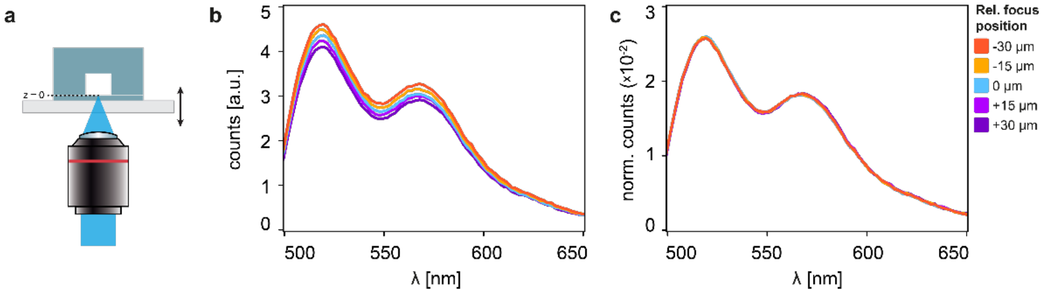

Fig. S5: Influence of focus position on FRET signal. (a) Schematic representation of focus point variation. (b) Background corrected FRET spectra for different focus position. (c) Normalized FRET spectra for the data presented in panel (b), demonstrating that the focus position only affects the SNR rather than the spectral FRET signature.

Buffer Sheath Flow

By introducing a buffer sheath flow, we prevent any reaction of the target with the analyte before the droplet has been formed. Fig. S6 presents an experimental observation of such a sheath flow with a clearly visible liquid phase boundary. In the case of three laminar flowing liquids (Fig. S6b) we only observe the phase boundary between the aptamer in 10% serum and the buffer phase (i.e. PBS). The phase boundary between buffer (PBS) and the serotonin (in PBS) is not visible due to the almost identical refractive index of the two liquids.

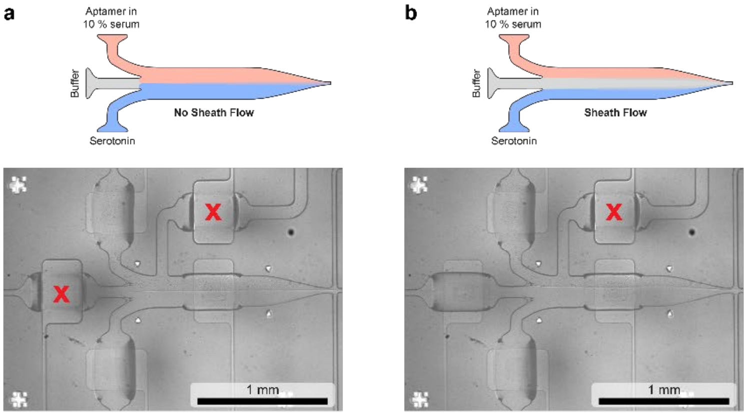

Fig. S6: Experimental observation of sheath flow. (a) Only two of the three inlet channels are open and equally pressurized. The resulting flow pattern allows the two reagents to interact at the interface. (b) By opening a third inlet, a buffer sheath flow is created and prevents any interaction between the actual reactants. For equal flow rates in all channels, the phase boundary shifts accordingly.

Read-out Principle for Droplet Speed

The droplet speed in [mm/s] is the product of droplet production frequency in [Hz] and the droplet separation in [mm]. The latter is observed from the big FoV camera whilst the droplet frequency is based on the APD time trace.

While the extraction of the droplet frequency is straight forward using a fast Fourier transformation (FFT), the read-out of the droplet interval is based on localizing the droplet positions within a straight channel segment. To do so, a line cut aligned with the channel is used to detect the edges of the droplets which then allows to calculate the droplet intervals under consideration of the magnification.

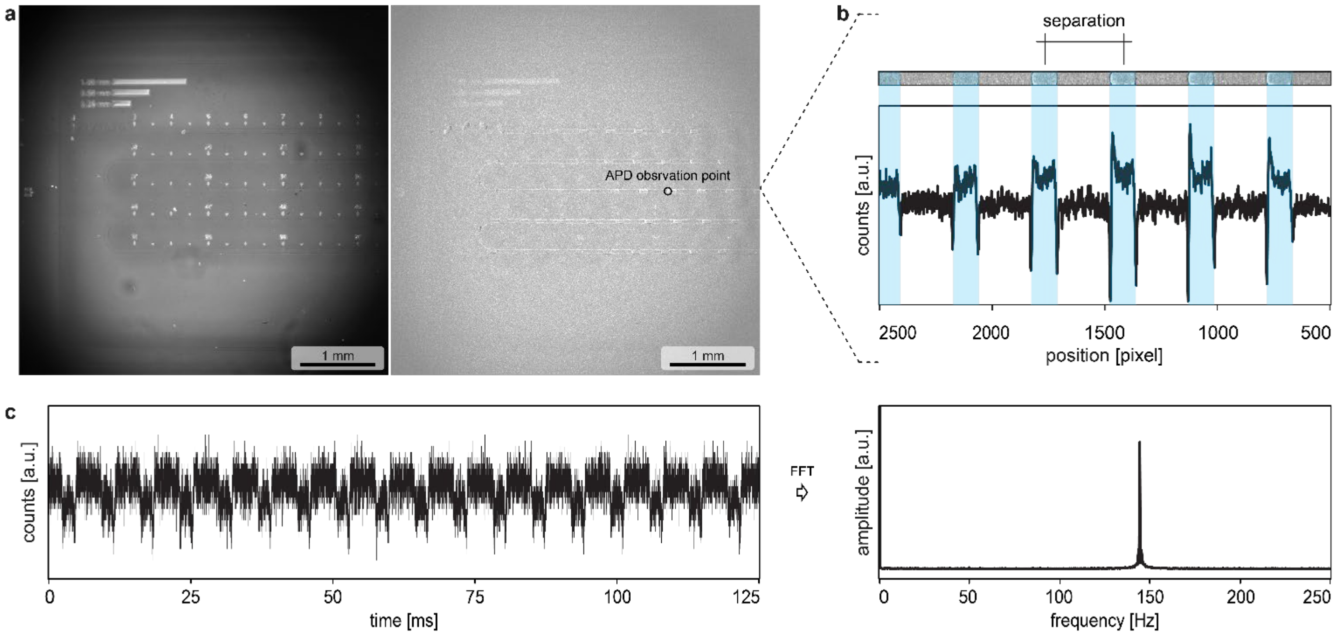

Fig. S7: Read-out of droplet velocity based on droplet interval and frequency. (a) Representative raw (left) and background subtracted (right) big FoV image used to read-out the droplet separation. (b) Exemplary line cut aligned with a segment of the outlet channel. (c) Representative time trace as recorded by the APD and corresponding FFT to extract the droplet production frequency.

Complementary Data based on ThT Assay for non-FRET Aptamers

As a complementary technique to the FRET measurements, a Thioflavin T (ThT) assay (Fig. S8) was performed. To do so, the oligonucleotides were first placed in boiling water for 5 minutes and then allowed to cool down to room temperature outside the water bath. Then the ThT dye solution was mixed with the aptamer in a 1:1 (v/v) ratio and incubated (protected from ambient light) for approximately 40 minutes. After that, the target solution at twice the concentration of the aptamer was added and the mixture was incubated again for at least 40 minutes. The final concentration of the aptamer and the ThT dye were 0.4 µM and 4 µM, respectively. The measurement (triplicates) was then carried out in 384-well black plates using a SpectraMax5 microplate reader (Molecular Devices, USA) with an excitation wavelength of 425 nm and an emission wavelength of 490 nm. The data as shown in Fig. S8, agrees in general with the FRET data – the aptamer with the shortest stem loop presents with the highest K_D and vice versa.

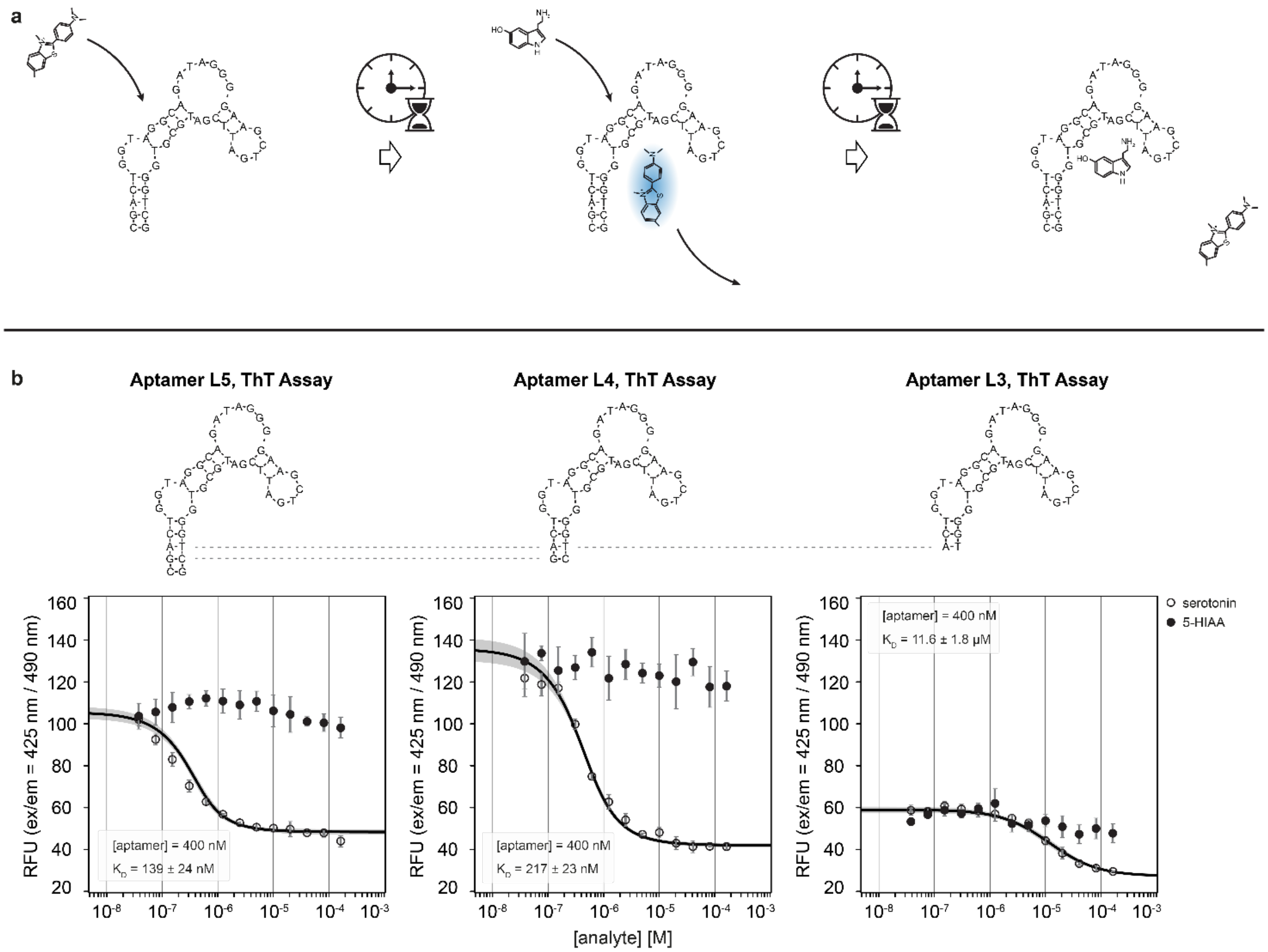

Fig. S8: Schematic representation of ThT assay principle and complementary ThT data. (a) Schematic representation of a ThT assay where the ThT dye and aptamer are mixed and incubated, allowing the dye to interact with the aptamer and become fluorescent upon excitation with a LASER. Next, the target molecule, here serotonin, is introduced and the mixture is incubated again, thereby, causing the displacement of the ThT dye. As the ThT dye is displaced by the target molecule, its fluorescence diminishes indicating the binding of the target molecule. (b) Complementary ThT assay data for the three investigated aptamers. The error bar represents the STD for three replicas. For the fit to a sigmoidal binding model the mean over the three replicas was used and weighted by the variance. The shaded area indicates a confidence interval of 90%. Also, note that the aptamer sequence is different by one nucleotide at the position of the former donor label which was FAMdT.

4-State Model for Kinetic Pathways of Serotonin Aptamer

Consistent with Lee et al.[1] we find two rate constants for the transition from high FRET to low FRET indicating hidden conformational transitions, inconsistent with a lock-and-key model of serotonin-aptamer binding. A 4-state model was found to be the best description of the kinetics, with two possible pathways to reach a stable, bound aptamer state. We define the model as state 1 (A+S, aptamer unbound state), states 2 and 3 ($AS^{‡}_1$ and $AS^{‡}_2$, intermediate bound conformations), and state 4 (AS). Transitions between the individual states are allowed as indicated in Fig. S9a. The nature of the ensemble measurement leaves the individual pathway information inaccessible; however, as an estimate we assign the observed kinetic rates from experiments to the pathway involving $AS^{‡}_1$ and describe the alternate pathway using scaled rate constants. The energy landscape from Lee et al. provides an estimate of the relative timescales between state transitions and is used for assigning a scaling factor M, as indicated in Fig. S9a. To preserve the detailed balance of the model, only one scaling factor for k_{31} and k_{34} is fit with additional scaling factors α and β for the forward and reverse reactions for the A+S—$AS^{‡}_2$—AS pathway. To calculate the population of each state over time a first-order linear differential equation, $\frac{dP}{dt} = \boldsymbol{Q}\vec{P}$, is used. Thereby, $\vec{P} = [\theta_A, \theta_{AS^‡_1}, \theta_{AS^‡_2}, \theta_{AS}]$ is the vector representing the probability fraction of the individual subpopulation at time t, with $\vec{P}_0$= [1, 0, 0, 0] indicating everything starts in the unbound state, and $\boldsymbol{Q}$ is the transition matrix:

$$\boldsymbol{Q} = \begin{bmatrix} -(k_{12}+k_{13}) & k_{21} & k_{31} & 0 \\ k_{12} & -(k_{21}+k_{24}) & 0 & k_{42} \\ k_{13} & 0 & -(k_{31}+k_{34}) & k_{43} \\ 0 & k_{24} & k_{34} & -(k_{42}+k_{43}) \end{bmatrix}$$

Given $\vec{P}$ at time t, the observed average $\langle E \rangle_{app.}$ is calculated as $\langle E \rangle_{app.} = \theta_A \langle E \rangle_A + \theta_{AS^‡_1} \langle E \rangle_{AS^‡_1} + \theta_{AS^‡_2} \langle E \rangle_{AS^‡_2} + \theta_{AS} \langle E \rangle_{AS}$, were $\langle E \rangle_A$, $\langle E \rangle_{AS^‡_1}$, $\langle E \rangle_{AS^‡_2}$ and $\langle E \rangle_{AS}$ are the subpopulation specific transfer efficiencies, of which only $\langle E \rangle_A$ and $\langle E \rangle_{AS}$ can be confidently be estimated from the experiments (in absence and with saturating concentration of serotonin respectively). For further calculation, similar to Lee et al.[1] We assumed that the two intermediate states $AS^{‡}_1$ and $AS^{‡}_2$ share the same transfer efficiencies of A+S and AS states respectively. Using this, a global fit of the experimental data was performed using scipy.integrate solve_ivp (method='RK45') and scipy.optimize least_squares (bounds = [0, 0], [inf, inf]; method='trf'; ftol=1e-8; xtol=1e-8) for the aptamers with stem loop length l = 5 and l = 4 individually. From this, values of M, α, and β were obtained, showing clear differences:

$M_{L=5,\ PBS} = 4.56 \pm 0.45$	$M_{L=5,\ Serum} = 1.53 \pm 0.16$	$M_{L=4,\ PBS} = 0.82 \pm 0.042$	$M_{L=4,\ Serum} = 0.60 \pm 0.064$
$\alpha_{L=5,\ PBS} = 0.41 \pm 0.016$	$\alpha_{L=5,\ Serum} = 1.03 \pm 0.078$	$\alpha_{L=4,\ PBS} = 0.98 \pm 0.015$	$\alpha_{L=4,\ Serum} = 1.086 \pm 0.024$
$\beta_{L=5,\ PBS} = 27.89 \pm 0.43$	$\beta_{L=5,\ Serum} = 37.69 \pm 1.41$	$\beta_{L=4,\ PBS} = 43.99 \pm 1.26$	$\beta_{L=4,\ Serum} = 64.14 \pm 6.05$

With the scaling factors extracted, the population of each state over time can be reconstructed.

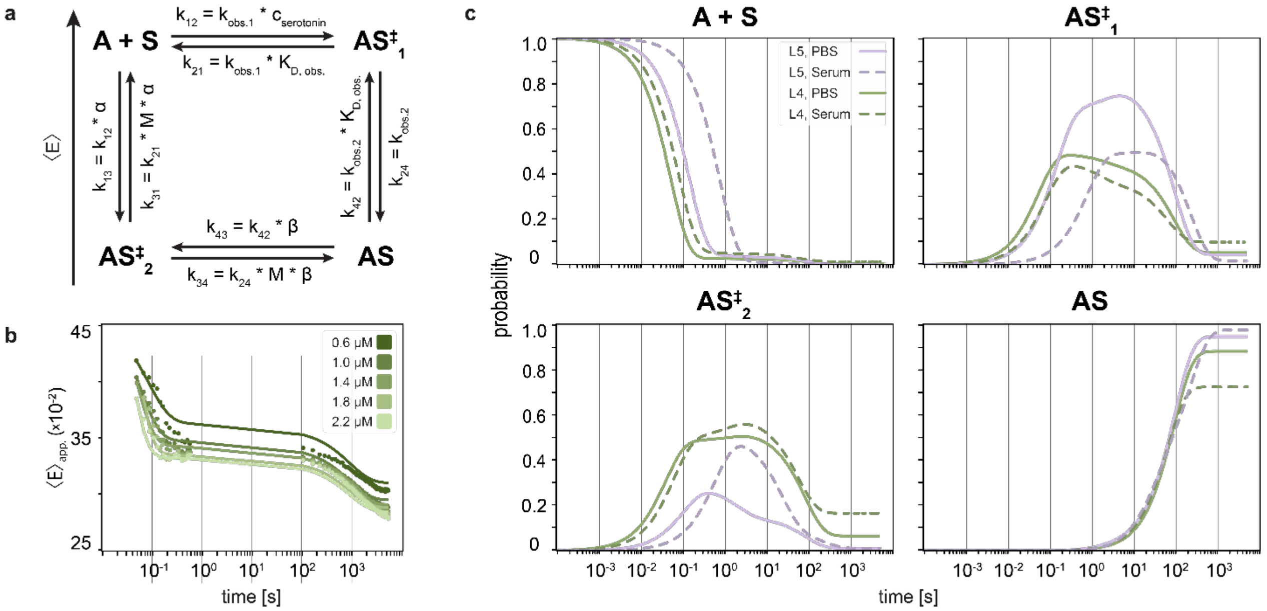

Fig. S9: 4-state model showing two different pathways for reaching the final bound aptamer-serotonin state. (a) Schematic of serotonin binding with four possible states, one high FRET (A+S), one low FRET (AS), and two intermediate bound states. (b) Fit of the binding model to the experimental data. Here shown for the aptamer L4 measured in PBS. (c) Calculated population of each state over time for aptamers L5 and L4 in PBS (solid line) and serum (dashed line).

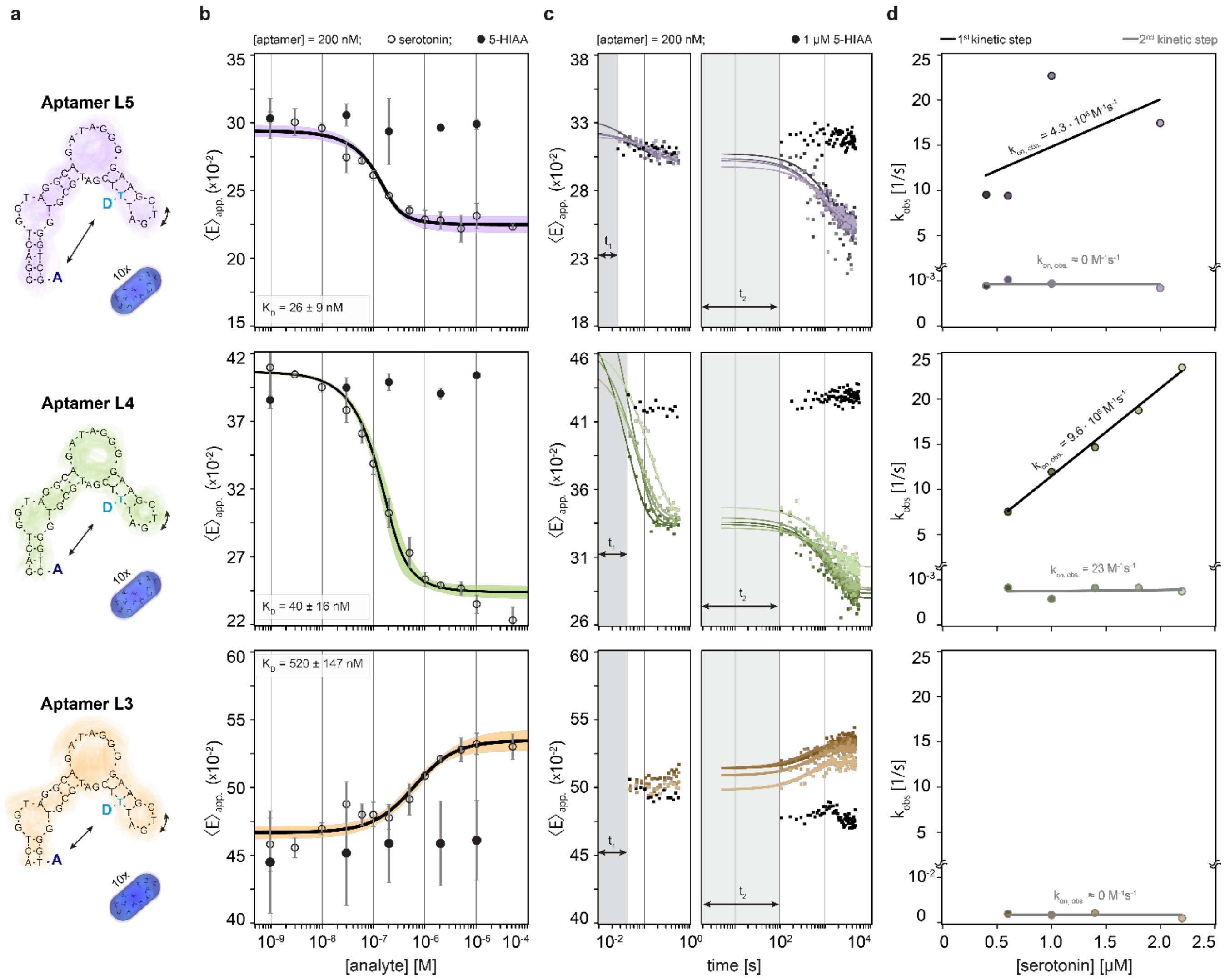

Fig. S10: Resolving different kinetic pathways based on 10 droplets averaged. (a) Schematic representation of the three investigated aptamers. (b) Equilibrium titration measurements in PBS after incubation. Error bars represent the STD for three replicas (different microfluidic chips). For the fit to a sigmoidal binding model the mean over the three replicas was used and weighted by the variance. The shaded area indicates a confidence interval of 90%. (c) Dynamic measurements for several concentrations of serotonin observed over short (ms) and long (min) time scales. Both time scales were individually fit to exponential models to extract the observed rate constants (k_{obs}) for each concentration on both time scales. t_1 and t_2 indicate the dead time related to in chip mixing and setting up the droplet experiment, respectively. (d) Plot of k_{obs} as a function of the serotonin concentration. The slope of the linear fit represents the on-rate of the aptamer for the specific kinetic state.

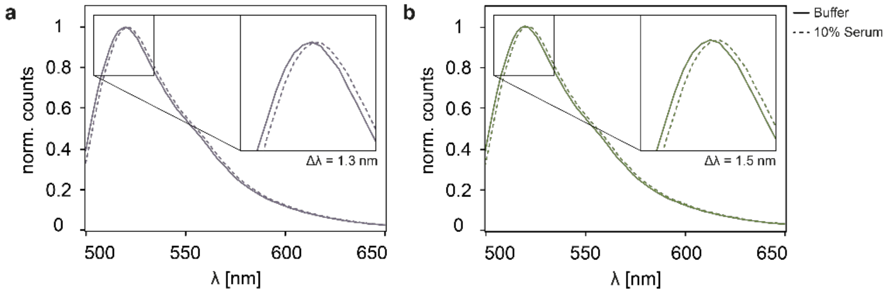

Fig. S11: Solvatochromic effect when changing from pure buffer to 10% human serum conditions. Shift of the donor emission peak for (a) aptamer L5 and (b) aptamer L4.

REFERENCES

(1) Lee, Y. *et al.* Carbon-nanotube field-effect transistors for resolving single-molecule aptamer–ligand binding kinetics. *Nat. Nanotechnol.* **2024**, *19,* 660–667.
